# A Mo_5_N_6_ electrocatalyst for efficient Na_2_S electrodeposition in room-temperature sodium-sulfur batteries

**DOI:** 10.1038/s41467-021-27551-7

**Published:** 2021-12-10

**Authors:** Chao Ye, Huanyu Jin, Jieqiong Shan, Yan Jiao, Huan Li, Qinfen Gu, Kenneth Davey, Haihui Wang, Shi-Zhang Qiao

**Affiliations:** 1 School of Chemical Engineering & Advanced Materials, The University of Adelaide, Adelaide, SA 5005 Australia; 2 Australian Synchrotron (ANSTO), 800 Blackburn Rd, Clayton, VIC 3168 Australia; 3 Beijing Key Laboratory of Membrane Materials and Engineering, Department of Chemical Engineering, Tsinghua University, 100084 Beijing, China

**Keywords:** Batteries, Electrocatalysis, Energy storage, Electrochemistry

## Abstract

Metal sulfides electrodeposition in sulfur cathodes mitigates the shuttle effect of polysulfides to achieve high Coulombic efficiency in secondary metal-sulfur batteries. However, fundamental understanding of metal sulfides electrodeposition and kinetics mechanism remains limited. Here using room-temperature sodium-sulfur cells as a model system, we report a Mo_5_N_6_ cathode material that enables efficient Na_2_S electrodeposition to achieve an initial discharge capacity of 512 mAh g^−1^ at a specific current of 1 675 mA g^−1^, and a final discharge capacity of 186 mAh g^−1^ after 10,000 cycles. Combined analyses from synchrotron-based spectroscopic characterizations, electrochemical kinetics measurements and density functional theory computations confirm that the high *d*-band position results in a low Na_2_S_2_ dissociation free energy for Mo_5_N_6_. This promotes Na_2_S electrodeposition, and thereby favours long-term cell cycling performance.

## Abstract (web summary)

Incomplete conversion of sodium polysulfides represents a significant issue in room-temperature sodium-sulfur batteries. Here, the authors propose Mo_5_N_6_ as an electrocatalyst for efficient Na_2_S electrodeposition and improved cell cycling performances.

## Introduction

Sulfur is an attractive electrode material because of low cost and high-theoretical specific capacity of ~1675 mAh g^−1^^1^. Sulfur electrodes can be conjugated with a range of metal anodes in rechargeable metal–sulfur (M–S) batteries, giving promise of practical energy-storage applications^2–4^. However, sulfur reduction reaction (SRR) in M–S batteries is a complex conversion from elemental sulfur to insoluble metal sulfides^5^. Sluggish SRR kinetics leads to incomplete conversion of the sulfur and “shuttle effect” of the polysulfides. This limits Coulombic efficiency (CE) and cycle-life and is therefore a deterrent to practical application^6^.

Metal sulfides electrodeposition from soluble polysulfides is the rate-determining step in practical sulfur electrodes^7^. Duan and co-workers demonstrated a slow conversion of soluble lithium polysulfides into insoluble lithium sulfides through studying the activation energy of various states of SRR in lithium–sulfur (Li–S) batteries^7^. This leads to accumulation of lithium polysulfides in the electrolyte, and is the primary reason for the shuttle effect, together with a rapid capacity fading^8^. Despite electronically conductive materials with active electrodeposition sites, such as heteroatom-doped carbon, typically applied as substrates to facilitate charge transfer, an atomic-level understanding of mechanism of metal sulfides electrodeposition in SRR is lacking^9–12^. For example, although it is known that Li_2_S electrodeposition is essential in Li–S batteries, the solid-solid conversion from Li_2_S_2_ to Li_2_S is unclear because it is difficult to distinguish various solid products in the complex process^13,14^. Moreover, the correlation between the electrodeposition kinetics and geometric/electronic structure of the cathode materials remains unknown^15–17^. Consequently, there is significant research interest in how to realize highly efficient metal sulfides electrodeposition in M–S batteries^18^.

Although SRR intermediates are too sensitive to be detected in air, advances in in-situ synchrotron characterizations with time resolution permit identification of specific polysulfides and metal sulfides and tracking of dynamic conversion^19–21^. In-depth understanding can therefore be achieved for macroscopic polysulfides conversion kinetics^22,23^. Nevertheless, atomic-level understanding of metal sulfides electrodeposition behavior is important and remains difficult to achieve experimentally^24^. Progress in density functional theory (DFT) computations that takes into account the geometric/electronic structure of the sulfur cathode materials is essential in investigating metal sulfides electrodeposition kinetics^25^. Therefore, combination of advanced in-situ synchrotron characterizations and computational quantum chemistry can reveal critical factors in metal sulfides electrodeposition kinetics^26,27^. Potential sulfur cathode materials with efficient metal sulfides electrodeposition kinetics can be engineered by tailoring geometric and electronic structures.

Here we present a Mo_5_N_6_ cathode material that significantly catalyzes Na_2_S electrodeposition and results in boosted performance for Na–S battery: 512 mAh g^−1^ capacity and long cycle life of 10,000 cycles under 1C (1675 mA g^−1^). Using a judicial combination of synchrotron-based characterizations, electrodeposition rate measurements and DFT computations, we evaluated the Mo_5_N_6_ catalysts by linking extrinsic geometric structure with intrinsic reaction energetics in the Na_2_S electrodeposition. With this research work we shed some light on the origin of high Na_2_S electrodeposition reactivity and high SRR efficiency of this cathode material.

## Results

### Atomic and electronic structures of molybdenum nitrides

**Fig. 1:**
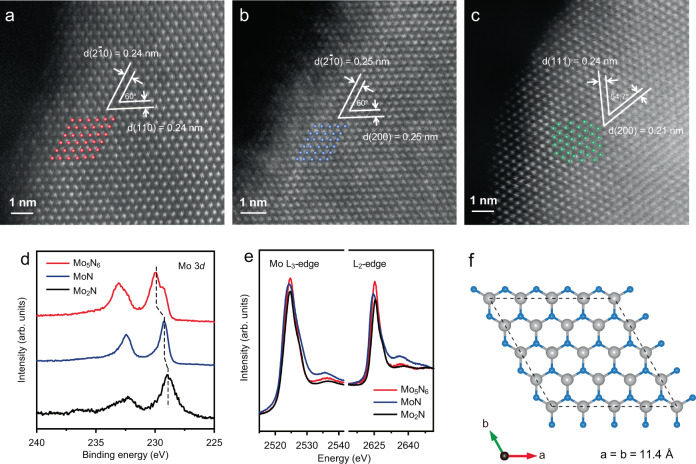
Atomic and electronic structural characterization of Mo_5_N_6_, MoN and Mo_2_N. **a**–**c** HAADF-STEM images of Mo_5_N_6_, MoN, and Mo_2_N. Insets are schematics of Mo configurations in which red-color, blue, and green spheres represent Mo atoms in Mo_5_N_6_, MoN and Mo_2_N, respectively. **d**, **e** Mo 3*d* XPS and Mo L_3_, L_2_-edge NEXAFS spectrum for Mo_5_N_6_, MoN, and Mo_2_N. **f** Computational model for Mo_5_N_6_ (0 0 4). The gray and light-blue spheres represent Mo and N atoms, respectively.

A series of molybdenum nitrides with varying atomic structure were selected as model cathode materials for investigation of correlation between the atomic structure, electronic structure and electrochemical performance in room-temperature sodium–sulfur (RT Na–S) batteries. Mo_5_N_6_, MoN, and Mo_2_N with varying stoichiometries were synthesized based on reported synthetic strategies^28^. The crystal phases of the as-prepared molybdenum nitrides were analysed by powder X-ray diffraction (XRD), and indexed to crystalline Mo_5_N_6_, MoN, and Mo_2_N, respectively (Supplementary Fig. 1). The scanning electron microscopy (SEM) images of these molybdenum nitrides confirm that the Mo_5_N_6_ and the MoN show a two-dimensional (2D) morphology and that Mo_2_N exhibits a homogeneous nanoparticle morphology (Supplementary Fig. 2). In addition, high-angle annular dark-field scanning transmission-electron microscopy (HAADF-STEM) imaging was performed to investigate the atomic structure of the molybdenum nitrides (Supplementary Fig. 3). As is shown in Fig. 1a, the Mo atoms are labeled as red-color spheres to aid visualization of the lattice fringes with a lattice distance of 0.24 nm, corresponding to the (2 $\bar{1}$ 0) and (1 1 0) facets of the Mo_5_N_6_^28^. In contrast, the MoN exhibits a lattice distance of 0.25 nm corresponding to the (200) and (2 $\bar{1}$ 0) facets. Mo_2_N has two lattice distances of 0.24 nm and 0.21 nm, which are ascribed to the (2 0 0) facet and (1 1 1) facet, respectively (Fig. 1b, c). This observation confirms the crystal structures of the molybdenum nitrides determined by XRD results and demonstrates the different Mo atomic configurations in the molybdenum nitrides.

It is reported that sulfur redox kinetics is significanty affected by the *d* electron density of the materials, which regulates atomic structure^29^. Mo 3*d* X-ray photoelectron spectra (XPS) measurements showed that the Mo_5_N_6_ has a higher dominant Mo valence state of 4+ with a binding energy of 230.0 eV, in contrast, the MoN and Mo_2_N exhibit lower binding energies of Mo species at 229.2 and 228.9 eV, respectively (Fig. 1d)^30^. This finding is supported by synchrotron-based near-edge X-ray absorption fine structure (NEXAFS) characterization that permits investigation of the impact on the surface electronic structures of the *d* electrons. The Mo L-edge white-lines originate from p electron transition to a vacant *d* electron state^31^. As is shown in the Mo-L_3_, L_2_ edge NEXAFS spectra (Fig. 1e), the intensity of adsorption edge peak decreases in the order Mo_5_N_6_, MoN, and Mo_2_N^32^. Therefore, the XPS and NEXAFS results demonstrate the high Mo valence state and low *d* electron density of Mo_5_N_6_ resulting from the unique atomic structure as is illustrated in Fig. 1f.

### Electrochemical properties of the molybdenum nitrides in the RT Na–S batteries

**Fig. 2:**
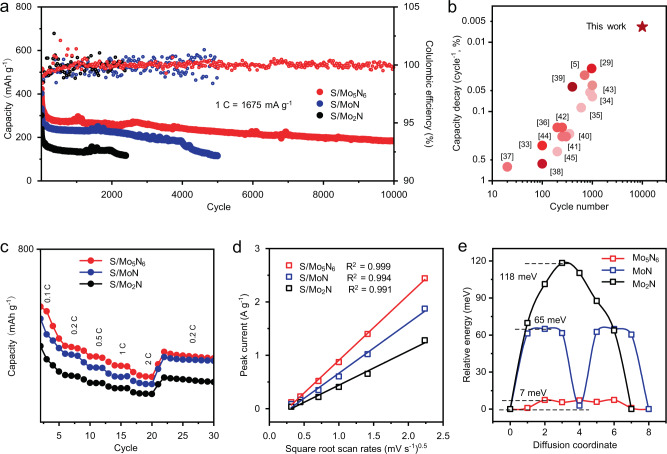
Electrochemical properties of the molybdenum nitrides in RT Na–S batteries. **a** Cycling performance and CE for the three sulfur electrodes at 1 C. **b** Comparison of cycle number and capacity retention of recently reported RT Na–S cells with the present work, in which darker-color refers to greater specific current. **c** Rating capacities for S/Mo_5_N_6_, S/MoN, and S/Mo_2_N. **d** Linear relationship between peak current (*i*_p_) and square root of scan rate (*v*^1/2^) for the three sulfur electrodes. **e** Energy profile for sodium ion diffusion on Mo_5_N_6_ (0 0 4) facet, MoN (0 0 2) facet, and Mo_2_N (1 0 0) facet.

To investigate electrocatalytic effects of molybdenum nitrides in RT Na–S batteries, we employed Mo_x_N_y_ catalysts as an additive and carbon–sulfur composite containing 62.9 wt% sulfur as active material in assembly of the sulfur electrodes. The as-prepared electrodes were denoted as S/Mo_5_N_6_, S/MoN, and S/Mo_2_N (Supplementary Fig. 4). For comparison, a pure S/C electrode was also prepared. Long-term cycling experiments at a high rate of 1C were conducted to investigate cycling performance of the sulfur electrodes (Fig. 2a and Supplementary Fig. 5). The capacity loss during initial few cycles was observed and is attributed to the formation of stable solid electrolyte interphase (SEI) film because of side effects between carbonated-based solvents and highly reactive sodium anode surface^23,33^. A high capacity of 186 mAh g^−1^ was maintained by the S/Mo_5_N_6_ after 10,000 continuous cycles under 1 C that refers to an capacity decay of 0.0064% per cycle, together with a stabilized CE held at around 100%. This performance significantly exceeds those of S/MoN (0.014%) and S/Mo_2_N (0.024%). In contrast, the S/C electrode exhibited a low initial capacity of 201 mA g^−1^ with a short cycle life of less than 200 cycles (Supplementary Fig. 6). Therefore, the S/Mo_5_N_6_ electrode has practical promise for a high-performance Na–S battery with high sulfur content, high capacity, small capacity decay and long cycling life in comparison with many reported sodium polysulfides cathodes, Na_2_S cathodes, or hybrid of carbon and sulfur cathodes in Na–S batteries (Fig. 2b and Supplementary Fig. 7 and Supplementary Table 1)^5,29,33–45^. As is shown in Fig. 2c, S/Mo_5_N_6_ exhibited a series of advantageous discharge capacities of 513, 353, 304, 263, and 216 mAh g^−1^ when cycled at 0.1, 0.2, 0.5, 1, and 2 C (1 C = 1675 mA g^−1^), respectively. When the specific current was switched back to 0.2 C, a high discharge capacity of 325 mAh g^−1^ was maintained (Supplementary Fig. 8). Over the following cycling at 0.2 C (Supplementary Figs. 9 and 10), the S/Mo_5_N_6_ exhibited a capacity decay of 0.013% per cycle (298–179 mAh g^−1^ in 2970 cycles). However, S/MoN, S/Mo_2_N and S/C cathodes show relatively poor cycling and rating performance with limited capacities under high rates (Supplementary Figs. 8–11).

To explore the origin of high CE and stability under high rates of the S/Mo_5_N_6_ cathode, we investigated the Na^+^ diffusion of the three sulfur cathodes through cyclic voltammetry (CV) experiments under various scan rates from 0.1 to 5 mV s^−1^ in the potential range 0.5–2.8 V^46^. During the initial cathodic scan (Supplementary Fig. 12) there was a prominent peak corresponding to reduction of elemental sulfur and long-chain soluble polysulfides to less soluble Na_2_S_2_ and Na_2_S. For the anodic scan, one reproducible peak was observed. This corresponds to the oxidation of Na_2_S_2_ and Na_2_S to Na_2_S_x_ and elemental sulfur^47^. The slow oxidation kinetics of Na_2_S_2_ and Na_2_S to Na_2_S_x_ are likely the cause for the overlapping of the two oxidation peaks during the anodic scan. Based on the experimentally obtained slope between the peak current (*i*_p_) and square root of the scan rate (*ν*^1/2^), the diffusion coefficient for sodium ions was estimated from: *i*_p_ ∝ *n*^3/2^AD^1/2^*cv*^1/2^, where *i*_p_, *n*, *D*, *A*, *c*, and *v* represent, respectively, peak current, number of electrons, diffusion coefficient, surface area of the electrode, concentration of the ion and voltage scanning rate (Fig. 2d)^48^. Because the number of electrons (*n*) and concentration of the sodium ion (*c*) are identical for the three sulfur electrodes, these cancel out in the *i*_p_-*ν*^1/2^ slope. Based on the electrochemical active surface areas (ECSAs) for the sulfur electrodes (Supplementary Fig. 13) the *i*_p_-*ν*^1/2^ slopes were normalized as is shown in Supplementary Fig. 14. It was confirmed that the S/Mo_5_N_6_ electrode exhibits greater sodium ion diffusion in comparison with that for S/MoN and S/Mo_2_N electrodes. This likely causes deposition of a thick Na_2_S layer on the electrode^49,50^.

To confirm the electrochemical measurements of diffusion rate, we computed the sodium ions diffusion barriers on the three molybdenum nitrides using climbing image nudged-elastic band (CI-NEB) method^21^. The three models were constructed based on the electron microscopy results, which are Mo_5_N_6_ exposing (0 0 4) facet, MoN with (0 0 2) facet, and Mo_2_N with (1 0 0) facet, as is shown in Supplementary Figure. 15^51^. The energy profiles for the ions diffusion on the three materials surface are shown in Fig. 2e, and the corresponding diffusion route in Supplementary Fig. 16. The diffusion barrier of sodium ions on the Mo_5_N_6_ (0 0 4) facet is 7 meV, which is significantly lower than those on the MoN (0 0 2) facet (65 meV) and Mo_2_N (1 0 0) facet (118 meV). Although the (0 0 4) facet for Mo_5_N_6_ and (0 0 2) facet for MoN can be regarded as the only primary exposed facets because of inherent 2D morphology, we considered other possible facets of Mo_2_N^52,53^. Given the 3D-morphology of Mo_2_N an additional major exposed facet of (1 1 1) was investigated. This showed a significantly greater sodium ion barrier of 246 meV over that for Mo_2_N (1 0 0) (Supplementary Figs. 17 and 18). Therefore, the computational results are consistent with the electrochemical results and confirm that the S/Mo_5_N_6_ electrode exhibits significantly greater sodium ion diffusion in comparison with the S/MoN and S/Mo_2_N electrodes. These findings demonstrate that the Mo_5_N_6_ represents an increased sodium ion diffusion rate and significantly promotes reaction kinetics between sodium and sulfur^54^.

### Kinetic investigations on Na_2_S electrodeposition

**Fig. 3:**
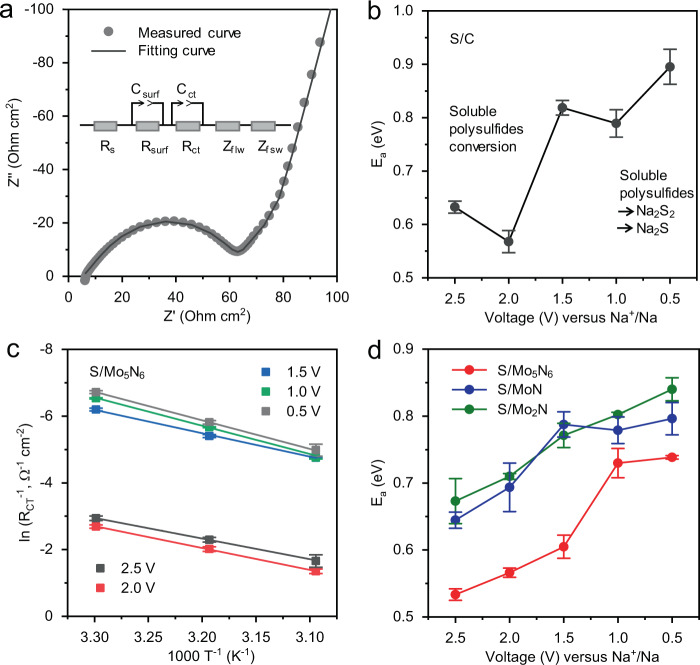
Overall kinetics of sulfur reduction reaction in RT Na–S cells. **a** Measured and fitted curve for S/C electrode. The measured EIS curve can be fitted with an equivalent circuit as is shown in the inset, in which *R*_surf_ describes deposition of adsorbed sodium polysulfides on the surface of the electrode, *R*_ct_ is attributed to the charge transfer process, and the tail line represents the Warburg resistance (*Z*_flw_ and *Z*_fsw_) in the pure sulfur cathode. **b** Activation energy for S/C electrode at varying voltage. The *E*_a_ study confirms that conversion of Na_2_S_4_ and/or Na_2_S_3_ to final insoluble Na_2_S_2_/Na_2_S is the rate-determining step for SRR. The errors originate from the linear fitting of Arrhenius plots for *R*_ct_. **c** Arrhenius-plots for *R*_ct_ for S/Mo_5_N_6_. Values for *R*_ct_ were obtained by fitting using an equivalent circuit as is shown in the inset of panel **a**. The error bars represent relative errors of the fitted *R*_ct_ values. **d** Activation energy for the three sulfur electrodes at varying voltage. Errors originate from the linear fitting of Arrhenius plots for *R*_ct_.

To determine the rate-determining step in overall SRR reaction, SRR kinetics were investigated on the S/C electrode via determination of the energy barrier (*E*_a_) with electrochemical impedance spectra (EIS) measurements and analysis^7^. The measured EIS curve was fitted with an equivalent circuit as is shown in Fig. 3a, in which *R*_surf_ describes deposition of the adsorbed sodium polysulfides on the surface of the electrode, *R*_ct_ is the charge transfer process, and the “tail” is the Warburg resistance (*Z*_flw_ and *Z*_fsw_)^55,56^. The EIS curves for the S/C electrodes under varying voltage were measured at temperatures of 303, 313, and 323 K (Supplementary Fig. 19). By fitting *R*_ct_ values in the Arrhenius equation, *E*_a_ at each voltage was evaluated (Supplementary Table 2). The conversion from S_8_ to Na_2_S_x_ (*x* = 5–8) at 2.5 and 2.0 V results in low *E*_a_ values of 0.63 and 0.57 eV, whilst the conversion following to Na_2_S_4_ and/or Na_2_S_3_ at 1.5 V exhibits an increased *E*_a_ of 0.82 eV (Fig. 3b and Supplementary Table 3). During final conversion to Na_2_S_2_/Na_2_S in the voltage range 1.0 to 0.5 V, *E*_a_ increases from 0.79 to 0.89 eV. These findings confirm that conversion of the S_8_ ring molecules to soluble Na_2_S_x_ is relatively facile, whereas the conversion of Na_2_S_4_ and/or Na_2_S_3_ to final insoluble Na_2_S_2_/Na_2_S is significantly more difficult, making it the rate-determining step for SRR. Similarly, EIS curves for S/Mo_5_N_6_, S/MoN, and S/Mo_2_N electrodes were measured at a temperature of 303, 313, and 323 K (Supplementary Figs. 20–22). As is shown in Fig. 3c, d, *E*_a_ for S/Mo_5_N_6_ under each of 2.5, 2.0, and 1.5 V, is, respectively, 0.53, 0.57, and 0.60 eV. These values are less than for S/MoN of, respectively, 0.64, 0.69, and 0.79 eV and for S/Mo_2_N of 0.67, 0.71, and 0.77 eV. Importantly, for the rate-determining step between 1.0 and 0.5 V, S/Mo_5_N_6_ exhibits significantly lower values of 0.73 and 0.74 eV in comparison with those for S/MoN of 0.78 and 0.80 eV, and for S/Mo_2_N of 0.80 and 0.84 eV. These findings evidence significantly boosted overall kinetics and Na_2_S electrodeposition on Mo_5_N_6_ compared with MoN and Mo_2_N.

**Fig. 4:**
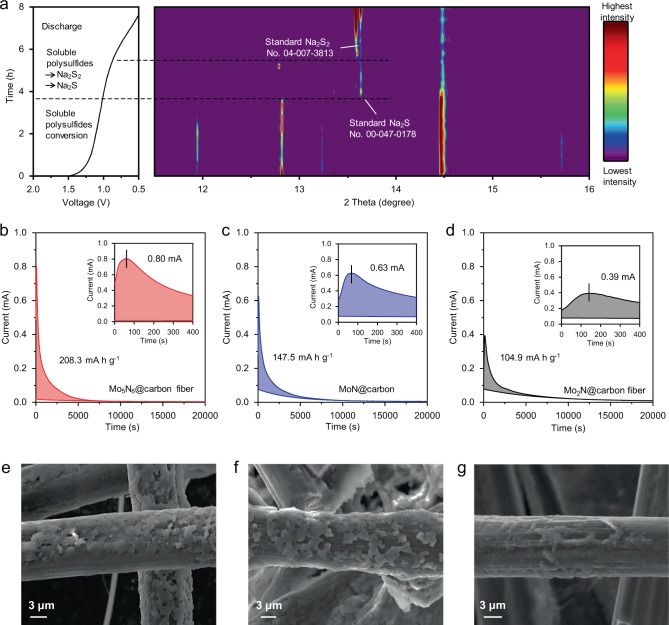
Kinetic investigations of Na_2_S electrodeposition. **a** Galvanostatic discharge curve and corresponding in-situ synchrotron XRD patterns for S/Mo_5_N_6_ electrode. **b**–**d** Potentiostatic discharge curves for electrodeposition rate measurements on the three materials. Insets are the first 400 s in the electrodeposition. **e**–**g** SEM images of electrodeposition tests on the three cathode materials.

To further investigate the Na_2_S electrodeposition kinetics on Mo_5_N_6_, in-situ synchrotron XRD measurements were conducted in transmission mode. A modified 2032-type coin cell with S/Mo_5_N_6_ as a cathode was discharged to 1.5 V (vs. Na^+^/Na) using an in-house design to study the Na_2_S electrodeposition, Fig. 4a (Supplementary Fig. 23)^20^. As can be seen from the figure in the discharge from 1.5 V, two peaks at 12.8° and 15.7° are evident in the XRD patterns. These are assigned to soluble Na_2_S_5_ (No. 00-027-0792)^5,37^. The other strong peak at 14.5° corresponds to Na_2_S_4_ (No. 04-003-2048). In addition, another peak at 11.9° is assigned to the Na_2_S_3_ (No. 00-044-0822)^5^. This finding reveals that the reduction of elemental sulfur to the mixture of sodium polysulfides occurs from open-circuit voltage to 1.5 V in discharge. This is consistent with the CV findings. When the battery was discharged to ~1.0 V, a peak at 13.6° was observed. This is attributed to the (4 0 4) facet of Na_2_S (No. 00-047-0178). In the further discharge to ~0.8 V, a peak located at 13.7° is assigned to the (1 0 2) facet of Na_2_S_2_ (No. 04-007-3813). In the discharge from 1.5 to 0.5 V, most of the polysulfides in the battery were oxidized to Na_2_S. Importantly, the peak for Na_2_S is seen before the appearance of the Na_2_S_2_ phase. This finding contrasts with previous report that Na_2_S_2_ was formed prior to Na_2_S^57^. This unusual phenomenon leads to an assumption that Mo_5_N_6_ exhibits very fast conversion kinetics from Na_2_S_2_ to Na_2_S, that result in highly favorable Na_2_S electrodeposition on the S/Mo_5_N_6_ cathode.

Na_2_S electrodeposition rate experiments were carried out to investigate electrodeposition kinetics of the three cathode materials. The potentiostatic experiments were designed and modified based on the reported Li_2_S electrodeposition experiments^11,58,59^. In order to distinguish the electrodeposition of Na_2_S from the reduction of higher-order polysulfides (Na_2_S_5_) in solution, the cell was discharged galvanostatically to a potential of 1.0 V at a rate of C/24. Subsequently, the electrodeposition was carried out at a fixed potential of 0.5 V (vs. Na/Na^+^). We present the fitting results via colored areas to demonstrate the capacity contributions of the Na_2_S electrodeposition in Fig. 4b–d. The capacities of the Na_2_S electrodeposition on carbon paper (CP)/Mo_5_N_6_, CP/MoN, and CP/Mo_2_N were determined to be 208.3, 147.5, and 104.9 mAh g^−1^ based on the sulfur mass, respectively. It is seen that Mo_5_N_6_ exhibits significantly greater Na_2_S electrodeposition capacity in comparison with that for MoN and Mo_2_N. In addition, the highest peak current of 0.80 mA was observed during the electrodeposition on the Mo_5_N_6_ as shown in the insets of Fig. 4b–d. The electrodeposition morphology of the Na_2_S on CP/Mo_5_N_6_, CP/MoN, CP/Mo_2_N, and CP was investigated by SEM imaging and energy-dispersive spectroscopy (EDS) mapping analysis (Fig. 4e–g and Supplementary Figs. 24–27). It was found that Na_2_S is uniformly deposited on the surface of CP/Mo_5_N_6_ with approximately 100% coverage. A three-dimensional (3D) deposition of Na_2_S layers on the CP/Mo_5_N_6_ highlights the high deposition efficiency^60^. This is attributed to the low energy barriers of Na_2_S nucleation and growth on Mo_5_N_6_. CP/MoN, CP/Mo_2_N, and CP exhibited an insufficient Na_2_S deposition with discrete coating as was evidenced by low Na_2_S coverage on the electrode surface (Supplementary Figs. 25–27). The sluggish Na_2_S electrodeposition kinetics on CP/MoN and CP/Mo_2_N are explained by the very high energy barriers for the redox reaction of polysulfides on them. The XPS spectra for the three sulfur electrodes discharged to 0.5 V were analyzed to confirm the electrochemical catalytic activity of Mo_5_N_6_. It is seen in Supplementary Fig. 28 that the S/Mo_5_N_6_ cathode exhibits dominate S^2−^ species at the end of discharge, underscoring a highly efficient conversion of active sulfur species to S^2−^^61,62^. In contrast, S/MoN and S/Mo_2_N cathodes show only partial conversion to S^2−^. This finding is attributed to the strong adsorption of sodium polysulfides and low diffusion barrier of sodium ions. The EIS tests were conducted at 0.5 V, and corresponding *R*_surf_ values which describe the deposition of insoluble sodium polysulfides on the electrode surfaces, and fitted to be ~485, ~281, and ~230 Ω cm^2^ for S/Mo_5_N_6_, S/MoN, and S/Mo_2_N electrodes, respectively (Supplementary Fig. 29). This finding agrees well with those from the Na_2_S electrodeposition test and the S 2p XPS analysis, confirming the highly efficient Na_2_S electrodeposition on Mo_5_N_6_ in comparison with the others^41,63^.

## Discussion

To determine the origin of the significant deposition efficiency and cycling stability of S/Mo_5_N_6_, DFT computations on thermodynamics and kinetics of Na_2_S electrodeposition on various molybdenum nitride surfaces were carried out. The adsorption configurations and adsorption energies for Na_2_S_n_ (*n* = 1–5) on the three nitrides were investigated. As is shown in the adsorption configurations, Supplementary Figs. 30–32, S in Na_2_S_n_ locates in the hollow sites on Mo_5_N_6_, whilst Na in Na_2_S_n_ locates away from the surface. This implies that Mo functions as a dominant adsorption site to interact with S in Na_2_S_n_^64^. The adsorption energies for Na_2_S_n_ on Mo_5_N_6_ were, respectively, −6.14, −8.75, −7.67, −6.36, and −9.23 eV for *n* = 1, 2, 3, 4, and 5, Supplementary Fig. 33 (Supplementary Table 4). These high values confirm strong adsorption of Na_2_S_n_ on Mo_5_N_6_ when compared with reported materials in sulfur cathodes for lithium/sodium polysulfides adsorption^65^. Although adsorption energies for Na_2_S_n_ on Mo_5_N_6_ were (slightly) greater than those on MoN, it was not clear why there is faster deposition kinetics on Mo_5_N_6_ compared with MoN.

**Fig. 5:**
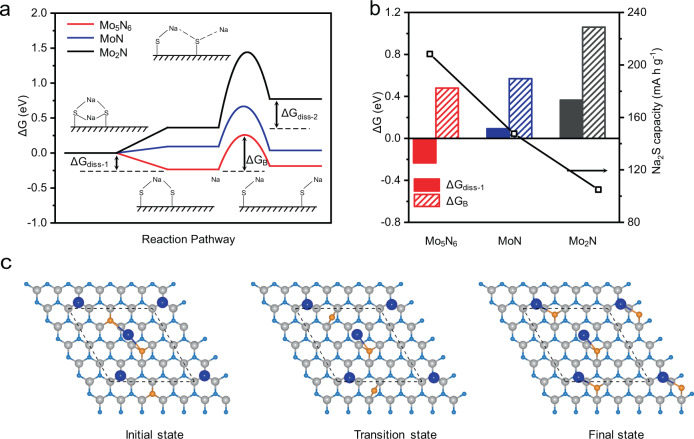
Computational investigation of Na_2_S electrodeposition. **a** Gibbs free energy diagram of conversion from Na_2_S_2_ to NaS* on the three surfaces including adsorption of Na_2_S_2_, dissociation of Na_2_S_2_ to form adsorbed NaS_2_ (NaS_2_${\,\!}^{\ast}$) and formation of adsorbed NaS (NaS^*^) from the NaS_2_^*^. Δ*G*_diss-1_ is dissociation free energy of Na_2_S_2_. Δ*G*_diss-2_ indicates formation free energy of NaS${\,\!}^{\ast}$, and Δ*G*_B_ indicates NaS^*^ formation free energy barrier. **b** Relationship between computed Δ*G*_diss-1_ or Δ*G*_B_ values and measured electrodeposition capacities on the three molybdenum nitrides surfaces. **c** Atomic configurations for NaS* formation step on the surface of Mo_5_N_6_. The gray-color, light blue, orange, and blue spheres represent Mo, N, S, and Na atoms, respectively.

To determine the origin of the fast Na_2_S electrodeposition kinetics on Mo_5_N_6_, we constructed a three-step reaction pathway for Na_2_S_2_ conversion to Na_2_S on the three molybdenum nitrides surfaces. The corresponding reaction steps are: 1) adsorption of Na_2_S_2_, 2) formation of adsorbed Na^*^ and NaS_2_^*^ from Na_2_S_2_ dissociation, 3) two NaS^*^ formation following simultaneous NaS_2_^*^ dissociation and a Na-S bond formation. For the Na_2_S_2_ dissociation step, Mo_5_N_6_ exhibits an optimal free energy (Δ*G*_Mo5N6 diss-1_) value of −0.23 eV, whilst MoN and Mo_2_N surfaces exhibit more positive values of Δ*G*_MoN diss-1_ = 0.09 eV and Δ*G*_Mo2N diss-1_ = 0.36 eV, respectively. However, for the NaS_2_^*^ dissociation step, MoN shows the optimal free energy (Δ*G*_MoN diss-2_) value of −0.05 eV, whilst Mo_5_N_6_ and Mo_2_N surfaces exhibit more positive values of Δ*G*_Mo5N6 diss-1_ = 0.04 eV and Δ*G*_Mo2N diss-1_ = 0.39 eV, respectively. Therefore, from a thermodynamic point of view, Mo_5_N_6_ and MoN demonstrate similar Na_2_S electrodeposition activity. However, these theoretical investigations based only on adsorption energetics do not agree with the experimental observation that CP/Mo_5_N_6_ sample demonstrates a significantly greater Na_2_S electrodeposition capacity in comparison with CP/MoN. When the kinetics of NaS^*^ formation step is considered (Fig. 5a and Supplementary Table 5), the Mo_5_N_6_ surface exhibits a substantially lower energy barrier (Δ*G*_B_ = 0.48 eV) than on MoN (Δ*G*_B_ = 0.58 eV) and Mo_2_N (Δ*G*_B_ = 1.06 eV) (Fig. 5b and Supplementary Figs. 34–36). Therefore, from a kinetic viewpoint, Mo_5_N_6_ demonstrates the most favorable electrodeposition efficiency amongst the three molybdenum nitride structures. With the identification of large energy barrier of NaS* formation on the Mo_2_N surface, in addition to the formation of the Na_2_S* state, the NaS* formation kinetics also affect the overall deposition rate and lead to the experimentally observed activity trend (Fig. 5c). Therefore, association of the atomic reaction model (applying Δ*G*_diss-1_ as a reactivity indicator) with the newly considered transition-state theory gives a qualitative confirmation of the deposition kinetics on the three molybdenum nitrides^66^.

**Fig. 6:**
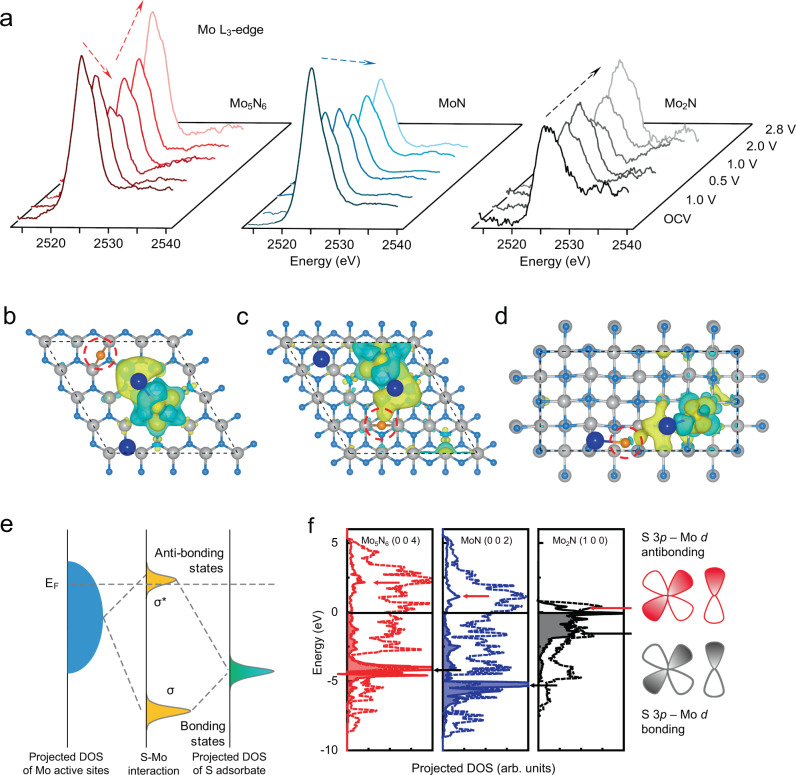
Analyzes of Mo electronic structure in corresponding reaction transition state. **a**–**c** Ex-situ NEXAFS for the Mo L_3_-edge of S/Mo_5_N_6_, S/MoN, and S/Mo_2_N, for varying voltage during a discharge/charge cycle. **b**–**d** Charge difference analyzes from configurations of the transition states in the NaS${\,\!}^{\ast}$ formation step on the three cathode materials, in which yellow-color and cyan iso-surface represent electron accumulation and electron depletion, respectively, and the iso-surface value is 0.0015 e Å^−3^. Color code is the same as for Fig. 5c. **e** Energy level diagram showing orbital hybridization for adsorption sites and adsorbate. *E*_F_ is the Fermi level of the substrate; *σ* and σ* indicate bonding and anti-bonding states, respectively. **f** Solid lines represent DOS for S in the red-color circles in Fig. 6b–d. The DOS is projected onto the S 3*p* state. Dashed lines represent surface Mo *d*-bands DOS of the three clean molybdenum nitrides surfaces.

To investigate the origin of fast Na_2_S electrodeposition kinetics on Mo_5_N_6_ from the aspect of electronic structure, spectroscopic measurements were carried out. The ex-situ NEXAFS characterizations were carried out on the three sulfur electrodes to investigate the dynamic change of the valence state of Mo in the three cathode materials. The Mo L_3_-edge spectra for Mo_5_N_6_ (Fig. 6a) show that the valence state of Mo decreases gradually with the discharge and reaches lowest level at the discharge potential of 0.5 V. This is consistent with the adsorption configuration from DFT results and demonstrates the strong Mo–S interaction and high Na_2_S electrodeposition capacity. During the following charge, the valence state of Mo increases to the original level until the end of charge at the potential of 2.8 V. This finding confirms the strong adsorption of sodium polysulfides and the high reversibility of Mo_5_N_6_ as electrode materials. As a comparison, similar measurements were performed on electrode with MoN. The NEXAFS spectra show that valence state of Mo decreases during discharge but remains unchanged during the following charge. This indicates that although MoN exhibits strong adsorption of sodium polysulfides, the high diffusion barrier of sodium ions on MoN leads to poor reversibility of the electrode. In contrast, the Mo_2_N electrode shows a nearly unchanged valence state of Mo during the whole discharge/charge. These findings confirm that Na_2_S electrodeposition kinetics on the molybdenum nitrides strongly depends on the valence state and d electron density of Mo.

To gain further fundamental insight into the low NaS_2_^*^ dissociation barrier on Mo_5_N_6_, charge difference analyses on the transition state configurations on three surfaces were carried out (Fig. 6b–d). On MoN and Mo_2_N surface, the interaction between the S (highlighted by red-color circles in Fig. 6c–d) and Na is strong, which represents electron accumulation between the two atoms. On the Mo_5_N_6_ surface, less electron accumulation is observed. This finding confirms a weaker interaction between the two atoms, which aids Na_2_S* dissociation with a lower dissociation barrier. The weaker interaction between Na and S is attributed to the strong Mo–S interaction, also demonstrated in the NEXAFS and XPS findings. The relationship between the adsorption of an adsorbate on a surface and the electronic structure of the substrate is explained by the density of states (DOS) (Fig. 6e). When a polysulfide molecule from the electrolyte is adsorbed on the molybdenum nitrides surface to form Na_2_S_n_*, the electronic states of the Mo interact with those of sulfur. Consequently, the hybridized energy levels split into two groups: one is the anti-bonding states (σ*) that normally go across the Fermi level (*E*_F_); the other is the bonding orbital (*σ*) positioned under the *E*_F_. The difference in the adsorption strength comes from the antibonding states, that is, with a higher location of the *E*_F_ of the molybdenum nitrides, the antibonding states move to lower occupancy. This leads to a stronger interaction between Na_2_S_n_* and the molybdenum nitrides surface, and vice versa^67^. In this work, the results of the DOS computations are consistent with the scheme, Fig. 6f. The low DOS peaks of the S 3*p*-Mo *d* antibonding orbitals (indicated by arrows) cause weak adsorption of S on MoN and Mo_2_N. More importantly, the position of antibonding orbital is decided by the *d*-band position of Mo on the molybdenum nitrides; the *d*-band centers for the Mo atoms on the Mo_5_N_6_, MoN, and Mo_2_N are −0.41, −1.56, and −2.41 eV, respectively. This is the same order as for the S–Mo antibonding peaks on the three surfaces. Therefore, the *d*-band position shows similar trends with the adsorption strength as is shown in Supplementary Fig. 37. The *d*-band center (applying as a descriptor) can be correlated with the Na_2_S_2_* dissociation energy as the underlying mechanism of the better electrodeposition reactivity of Mo_5_N_6_ in sulfur cathodes.

Using sodium-sulfur chemistry as an example, we correlated the Na_2_S electrodeposition reactivity on a Mo_5_N_6_ electrocatalyst with its reaction energetics and inherent electronic structure. By identifying the significant influence of Na_2_S_2_ dissociation on the overall SRR reactivity of various molybdenum nitrides surfaces, we elucidated the mechanism of metal sulfides electrodeposition through the association of atomic reaction model with the transition-state theory. The combination of experimental data and theoretical computations demonstrates that Mo_5_N_6_ with favorable *d*-band position delivers significantly high Na_2_S electrodeposition reactivity and performance in Na-S battery. This advance in mechanistic understanding of metal sulfides electrodeposition will underpin rational design of efficient M–S batteries. Application has resulted in significant performance of an RT Na–S battery. Findings will be of immediate interest and practical benefit to a wide range of researchers in the rational design of electrode materials for accelerated applications in sustainable energy-storage and conversion.

## Methods

### Preparation of molybdenum nitrides

Mo powder (<150 μm, 99.99% trace metals basis) was purchased from Sigma-Aldrich without further purification. The 2D Mo_5_N_6_ nanosheets were synthesized through a Ni-induced salt-templated method as previously reported: 0.4 g of Mo powder was dispersed in 40 mL of ethanol with magnetic stirring for 10 min^28^. 1.2 mL of H_2_O_2_ (30 wt%) solution was injected dropwise into the suspension. Following stirring for 12 h at room temperature, the solution turned into a dark-blue color. Separately, 10 mg of Ni(OCOCH_3_)_2_·4H_2_O was dissolved in 10 mL of ethanol and mixed with the dark-blue suspension to form the precursor. The precursor solution was mixed with 640 g of NaCl powder and dried at 50 °C with continuous hand-stirring. The mixture was annealed at 750 °C for 5 h at a heating rate of 1 °C min^−1^ under a 5% NH_3_/Ar atmosphere. The product was washed with deionized water and dilute hydrochloric acid several times to remove the NaCl template and Ni nanoparticles before being dried using vacuum filtration. The 2D MoN nanosheets were synthesized without addition of Ni. The Mo precursor was mixed with 640 g of NaCl and annealed at 750 °C for 5 h. The final product was obtained by removing NaCl using deionized water and vacuum filtration. The Mo_2_N nanoparticles were produced by annealing Mo powder under NH_3_ atmosphere. Fifty milligram of Mo powder was put into a porcelain boat uniformly. The powder was annealed at 650 °C for 5 h at the ramp rate of 1 °C min^−1^ under 5% NH_3_/Ar atmosphere.

### Materials characterization

The morphology and structure of samples was characterized by SEM (FEI Quanta 450). HAADF-STEM images were recorded at 200 kV (Talos F200X). XRD data were recorded on a Rigaku MiniFlex 600 X-Ray Diffractometer. Sulfur content of the active material was determined by TGA (METTLER TOLEDO TGA/DSC 2) under N_2_. In-situ synchrotron XRD (with wavelength *λ* = 0.6888 Å) and NEXAFS data were detected on the powder diffraction and the soft X-ray spectroscopy beamline in the Australian Synchrotron, Clayton, Victoria.

### Electrochemical characterization

For the battery performance measurement, active sulfur material containing elemental sulfur and conductive carbon (Ketjen Balck) with mass ratio of 2:1 was well-mixed and sealed in a quartz ampoule and thermally treated at 300 °C for 2 h under a N_2_ atmosphere. 5 wt% Mo_x_N_y_ was used as the additive in the slurry together with 80 wt% of active material, 5 wt% of conductive carbon and 10 wt% N-lauryl acrylate (LA133, purchased from Chengdu Yindile Power Supply Technology). For comparison, a pure S/C electrode was prepared with a slurry containing 80 wt% active material, 10 wt% conductive carbon and 10 wt% N-lauryl acrylate. The slurry mixture was cast on aluminum-foil and dried at 50 °C overnight to fabricate the sulfur electrodes with average thickness of ~20 μm. The 2032-type coin cells were assembled using glass-fiber as the separator and Na metal with average thickness of ~500 μm and purity of 99.9% as the anode. The electrolyte consisted of 1.0 M NaClO_4_ in ethylene carbonate (EC)/propylene carbonate (PC) with a volume ratio of 1:1 and 5 wt% fluoroethylene carbonate (FEC) additive. The volume of electrolyte injected into the coin cells was controlled to 15 μL in total. The areal active material loading in the cathode for rating and cycling performance was ~1.2 mg cm^−2^. The area of the sulfur electrodes are ~1.13 cm^−2^. The galvanostatic charge/discharge measurements were performed under 30 °C in a constant temperature oven using NEWARE and LAND CT2001A battery testers. The capacities were calculated based on the mass of the elemental sulfur. For all the cycling and rating tests, a 0.1 C low-rate cycle were adopted to activate the redox reactivity of the elemental sulfur. Electrochemical impedance spectroscopy tests were performed at specific voltages in the frequency range 1 MHz to 0.01 Hz with an amplitude of 5 mV after resting for 10 min. A thermal test chamber (MSK-TE906) was used to control the temperature during the EIS testing. To stabilize voltage, the battery was discharged to the particular potential and held at that potential until the output current remained constant. For in-situ synchrotron XRD measurements, the in-situ cells were similar to the coin cells for electrochemical performance testing and were discharged to 1.5 V under 0.1 C prior to measurement of Na_2_S electrodeposition. To guarantee that the X-ray beams could penetrate the whole cell, three holes with 4 mm diameter were punched in the center of the negative, the positive battery shells and the spacer. Kapton film was used to cover the holes in the negative and positive caps, and glue was used for sealing. The assembly of the in-situ cells was the same as that for the cells for the electrochemical tests. For the ex-situ measurements, the cells with test electrodes were discharged or charged to the specific voltage under 0.1 C, then were dissembled in a glove box. The test electrodes were sealed in a sample-holder within the glove box to transport the electrode samples.

### Na_2_S electrodeposition experiments

The Na_2_S (>97.0%), sulfur powder (reagent grade, 100 mesh particle size), tetraglyme (>99%) and glass fiber separator (Whatman® glass microfiber filter, Grade GF/F) were purchased from Sigma-Aldrich Australia. The Na_2_S powder was stored in glove box. The carbon papers (CF) were purchased from Shanghai Hesen Electronics Co. Ltd. (HCP010N). The original Li_2_S electrodeposition tests are referred to for the Na_2_S electrodeposition tests^11^. The Na_2_S electrodeposition from soluble polysulfides was studied by potentiostatic deposition in Na_2_S_4_ tetraglyme solution on the CF current collectors. The Na_2_S_4_ tetraglyme solution was applied to compare the delicate kinetical differences for different samples with corresponding electrodeposition films and capacities. The test was conducted in a similar coin cell as for the electrochemical performance tests. The Na_2_S_4_ in tetraglyme solution added to the Mo_x_N_y_ on CF was the only source of sulfur active material. The 0.1 M Na_2_S_4_ solution was prepared by dissolving and mixing stoichiometric amounts of Na_2_S and sulfur in tetraglyme solvent at room temperature for 10 h. CF papers were punched into disks with a diameter of 12 mm and about 0.40 mg of Mo_5_N_6_, MoN, and Mo_2_N powders were separately dispersed on CF papers using pure ethanol as solvent. 25 µL Na_2_S_4_ was dropped onto the as-prepared current collectors as cathode. Sodium-foil was employed at the counter electrode, which was separated with cathode by glassfiber membrane and dropped with 15 µL electrolyte. The cells were galvanostatically discharged to 1.0 V at a constant specific current of C/24, and kept potentiostatically at 0.5 V for Na_2_S to nucleate and grow until the current dropped below 10^−5^ A.

### Computational methods

DFT computations were carried out using the Vienna Ab-initio Simulation Package (VASP)^68,69^. The exchange-correlation interaction was described by generalized gradient approximation (GGA) with the Perdew–Burke–Ernzerhof (PBE) functional^70^. The DFT-TS method of Grimme was employed to treat the VDW interaction^71^. All computations were carried out using a plane wave kinetic energy cut-off of 600 eV. All structures in the computations were spin-polarized and relaxed until the convergence tolerance of force on each atom was less than 0.01 eV. The energy convergence criteria were set to 10^−4^ eV for self-consistent computations with a Gamma centered 2 × 2 × 1 K-points. All periodic slabs had a vacuum spacing of at least 15 Å. Denser 10 × 10 × 1 K-points were used for the density of states (DOS) computations. All periodic slabs had a vacuum spacing of at least 15 Å. The structural model for Mo_5_N_6_ (0 0 4) facet contained five Mo-N layers with a supercell size of *a* = *b* = 11.44 Å, *c* = 20.60 Å, *α* = *β* = 90°, and *γ* = 120°. The structural model for MoN (0 0 2) facet contains five Mo–N layers with a supercell size of *a* = *b* = 11.36 Å, *c* = 20.70 Å, *α* = *β* = 90° and *γ* = 120° while the Mo_2_N (1 0 0) facet consisted of five Mo–N layers with *a* = *b* = 8.33 Å, *c* = 23.33 Å, *α* = *β* = *γ* = 90°. In computations the three bottom layers were kept fixed, all other atoms were allowed to relax. Na_2_S_n_ (*n* = 1–5) adsorption energies Δ*E* for each configuration were computed from:

1 $$\varDelta E={E}_{{{{{{\mathrm{total}}}}}}}-{E}_{{{{{{\mathrm{Na2Sn}}}}}}}-{E}_{{{{{{\mathrm{s}}}}}}}$$

where *E*_total_, *E*_Na2Sn_, and *E*_s_ are, respectively, the energies for the whole system, Na_2_S_n_ and substrate.

### Free energy diagram computation

Based on the experimentally obtained rate-determining step, the solid-solid conversion from Na_2_S_2_ to Na_2_S was proposed as three elementary steps, namely:

2a $$\ast {{{{{{\rm{Na}}}}}}}_{2}{{{{{{\rm{S}}}}}}}_{2}+2{{{{{{\rm{Na}}}}}}}^{+}+2{{{{{{\rm{e}}}}}}}^{-}$$

2b $$\ast {{{{{{\rm{NaS}}}}}}}_{2}+\ast {{{{{\rm{Na}}}}}}+2{{{{{{\rm{Na}}}}}}}^{+}+2{{{{{{\rm{e}}}}}}}^{-}$$

2c $$2\ast {{{{{\rm{NaS}}}}}}+2{{{{{{\rm{Na}}}}}}}^{+}+2{{{{{{\rm{e}}}}}}}^{-}$$

in which * indicates a reaction site. The free energies for the three steps are Δ*G*_diss-1_ and Δ*G*_diss-2_, respectively, and can be computed from:

3 $$\varDelta {{{{{{\rm{G}}}}}}}_{{{{{{\rm{diss}}}}}}-1}={{{{{\rm{G}}}}}}(\ast {{{{{{\rm{NaS}}}}}}}_{2}+\ast {{{{{\rm{Na}}}}}})-{{{{{\rm{G}}}}}}(\ast {{{{{{\rm{Na}}}}}}}_{2}{{{{{{\rm{S}}}}}}}_{2})$$

4 $$\varDelta {{{{{{\rm{G}}}}}}}_{{{{{{\rm{diss}}}}}}-2}={{{{{\rm{G}}}}}}(2\ast {{{{{\rm{NaS}}}}}})-{{{{{\rm{G}}}}}}(\ast {{{{{{\rm{NaS}}}}}}}_{2}+\ast {{{{{\rm{Na}}}}}})$$

in which

5 $${{G}}={{E}}+{{{E}}}_{{{{{{\rm{ZPE}}}}}}}-{{{{{\rm{TS}}}}}}$$

and where zero point energy corrections (*E*_ZPE_) and entropic contributions (TS; *T* was set to be 273.15 K). The free energy of the Δ*G*_diss-1_ and Δ*G*_diss-2_ were computed as:

6 $$\Delta {{G}}=\Delta {{E}}+\Delta {{{E}}}_{{{{{{\rm{ZPE}}}}}}}-{{T}}\Delta {{S}},$$

where Δ*E*_ZPE_ and Δ*S* are the binding energy, zero point energy change and entropy change, respectively. In this work the values of Δ*E*_ZPE_ and Δ*S* on the specific molybdenum nitrides surfaces were determined by vibrational frequency computation. Note that the exploration of active sites on the specific surface was conducted and chosen according to the most energetically stable adsorption site.

### Na_2_S_2_ dissociation barrier computation

Following identification of the initial and final states for Na_2_S_2_ dissociation step on the three molybdenum nitrides surfaces, the energy barrier was located via searching for transition states by climbing image nudged-elastic band (CI-NEB) method implemented in VASP^21^. The transition states were obtained by relaxing the force below 0.05 eV Å^−1^. The located transition states were confirmed by frequency analysis. The *d*-band center ($\varepsilon$_d_) was determined as the weighted DOS center of *d*-band as:

7 $${\varepsilon }_{{{{{{\rm{d}}}}}}}={\sum }_{{{{{{\mathrm{i}}}}}}}{\varepsilon }_{{{{{{\mathrm{i}}}}}}}{{{{{{\rm{r}}}}}}}_{{{{{{\mathrm{i}}}}}}}/{\sum }_{{{{{{\mathrm{i}}}}}}}{\varepsilon }_{{{{{{\mathrm{i}}}}}}}$$

where *r*_i_ is the DOS at energy $\varepsilon$_i_.

### Reporting summary

Further information on research design is available in the Nature Research Reporting Summary linked to this article.

## Supplementary information

Supplementary Information

Reporting Summary

## Data Availability

Data that support findings from this study are available from the corresponding author on request.
