# Supplementary material for: A Mo5N6 electrocatalyst for efficient Na2S electrodeposition in room-temperature sodium-sulfur batteries
Source: Nat Commun. 2021 Dec 10;12:7195. doi: 10.1038/s41467-021-27551-7 (PMC8664834; doi:10.1038/s41467-021-27551-7)
Supplement: Supplementary file 1 — Supplementary Information [file 41467_2021_27551_MOESM1_ESM.pdf]

A Mo_5N_6 Electrocatalyst for Efficient Na_2S Electrodeposition in Room-Temperature Sodium-Sulfur Batteries

Chao Ye[1†], Huanyu Jin[1†], Jieqiong Shan[1†], Yan Jiao[1], Huan Li[1], Qinfen Gu[2], Kenneth Davey[1], Haihui Wang[3,*], Shi-Zhang Qiao[1,*]

[1] School of Chemical Engineering & Advanced Materials, The University of Adelaide, Adelaide, SA 5005, Australia.

[2] Australian Synchrotron (ANSTO), 800 Blackburn Rd, Clayton, VIC 3168, Australia.

[3] Beijing Key Laboratory of Membrane Materials and Engineering, Department of Chemical Engineering, Tsinghua University, Beijing, 100084, China.

†These authors contributed equally to this work.

*Email: s.qiao@adelaide.edu.au ; cehhwang@tsinghua.edu.cn

Supplementary Figures

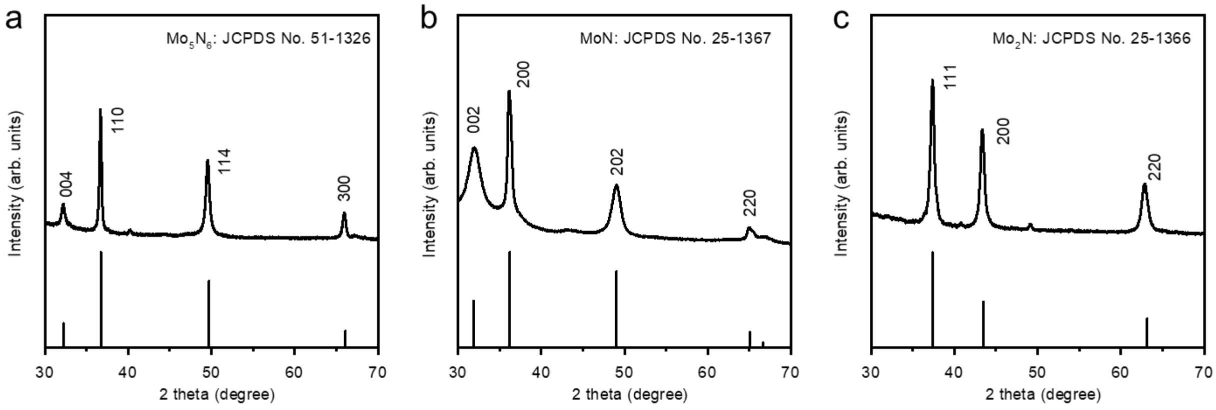

Supplementary Figure 1. a-c, XRD patterns for Mo_5N_6, MoN and Mo_2N.

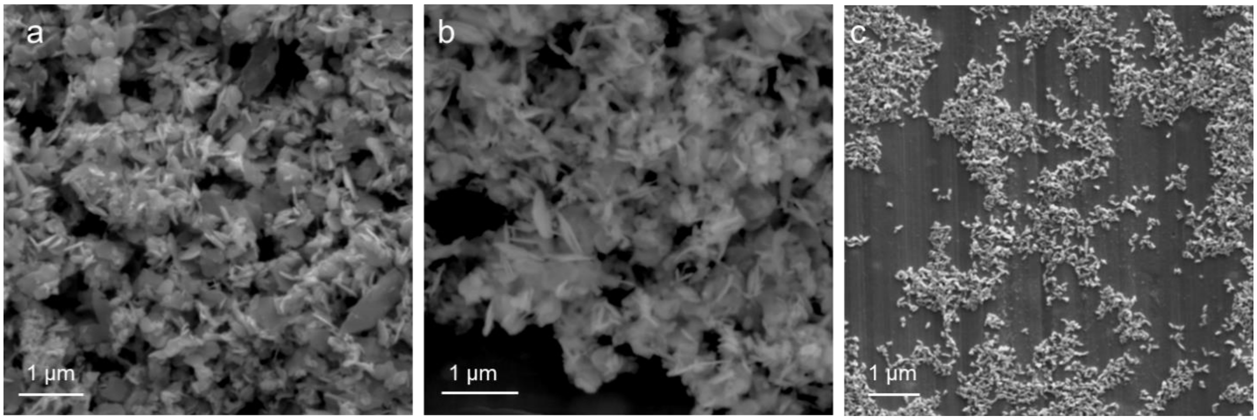

Supplementary Figure 2. a-c, SEM images of Mo_5N_6, MoN and Mo_2N.

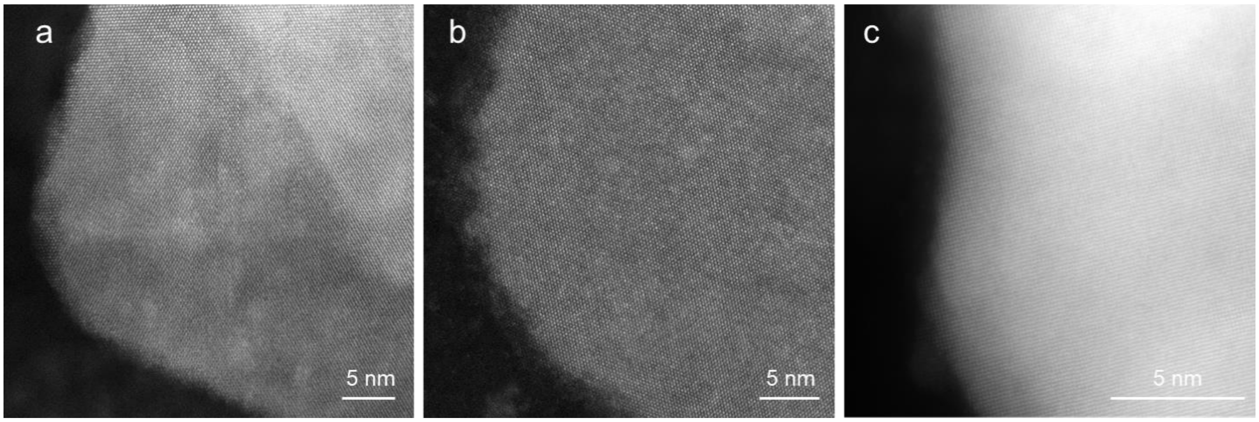

Supplementary Figure 3. a-c, HAADF-STEM images of Mo_5N_6, MoN and Mo_2N.

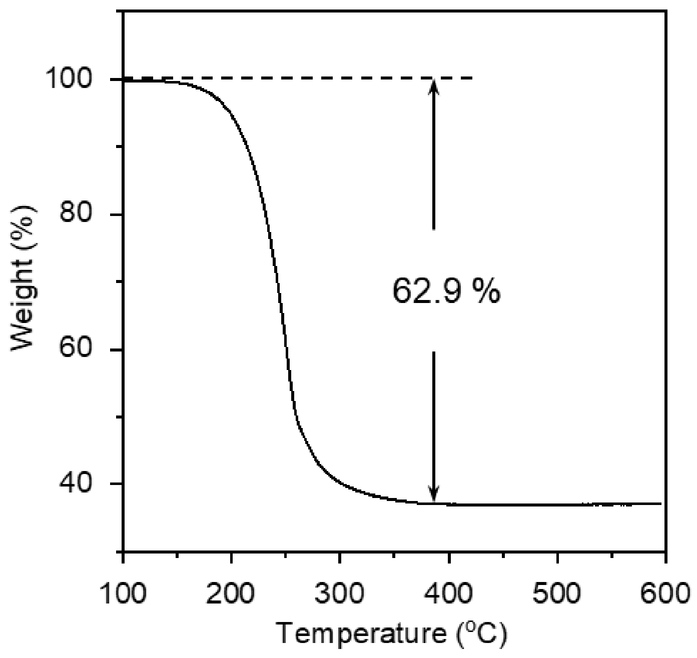

Supplementary Figure 4. TGA curve for the active sulfur material.

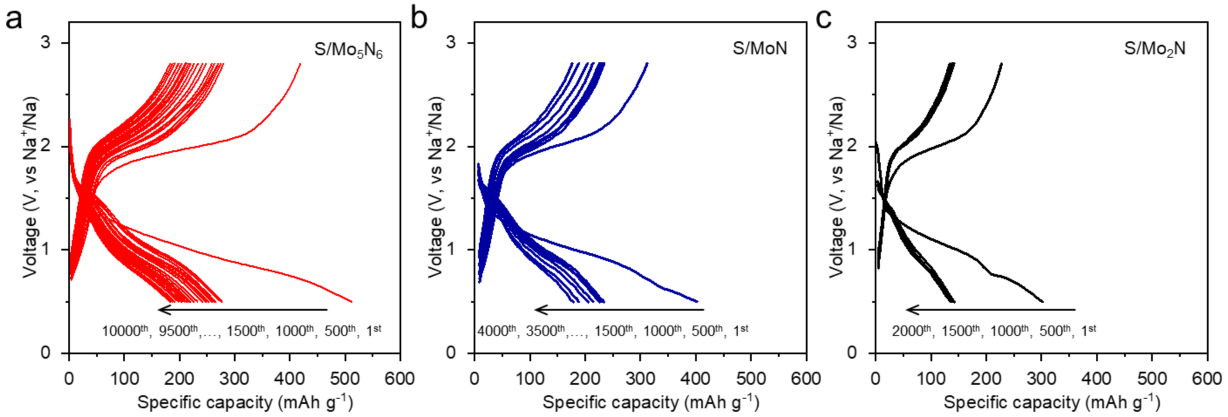

Supplementary Figure 5. Discharge/charge curves for **a,** S/Mo_5N_6, **b,** S/MoN and **c,** S/Mo_2N electrodes during cycling test at 1 C.

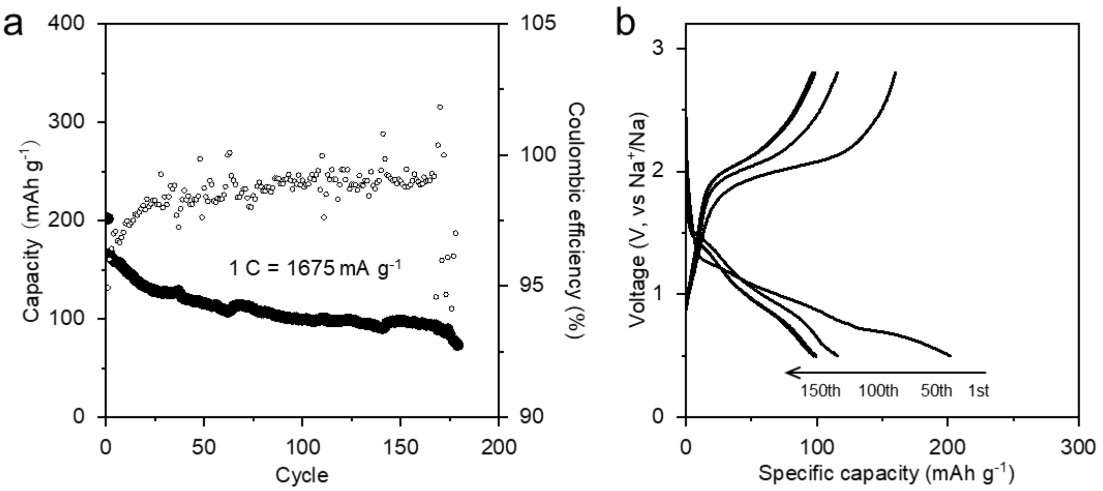

Supplementary Figure 6. a, Cycling performance and CE of the S/C electrode at 1 C. **b,** Discharge/charge curves of the S/C electrode at 1 C.

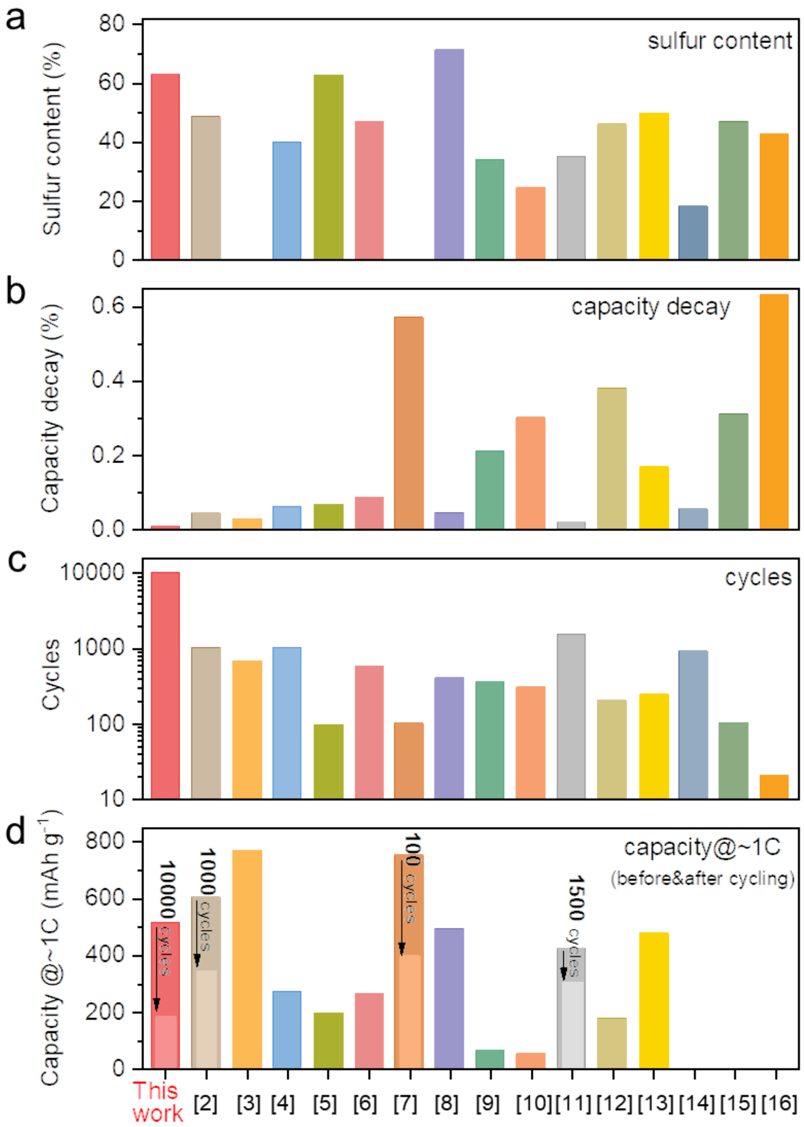

Supplementary Figure 7. Performance comparison of S/Mo_5N_6 and recently reported Na-S cell cathode materials in terms of **a,** sulfur content; **b,** capacity decay over cycling test; **c,** cycle numbers and **d,** capacity under specific current of around 1 C. In panel d, the overlaying bars indicate the capacities before and following cycling test, while the other bars demonstrate the capacities obtained in rate test at around 1 C.

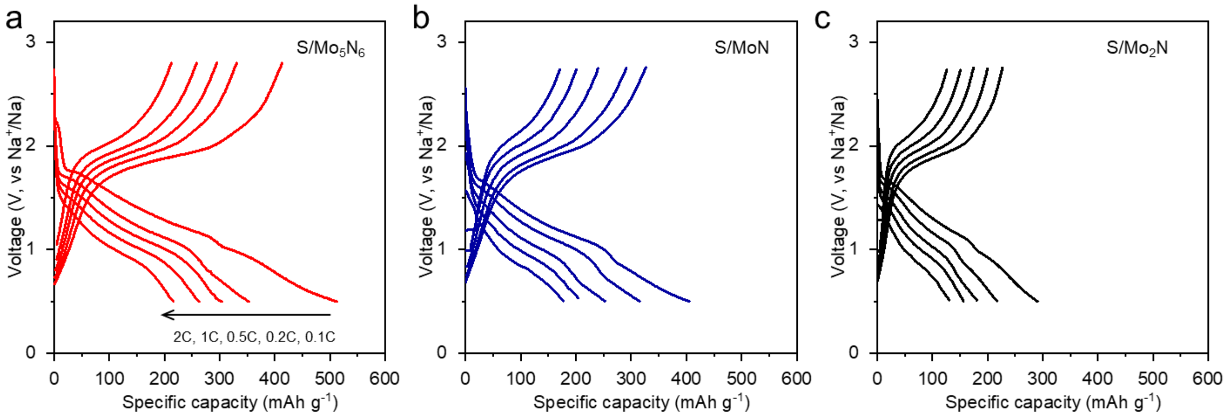

Supplementary Figure 8. a-c, Discharge/charge curves of the three sulfur electrodes under rates from 0.1 to 2 C.

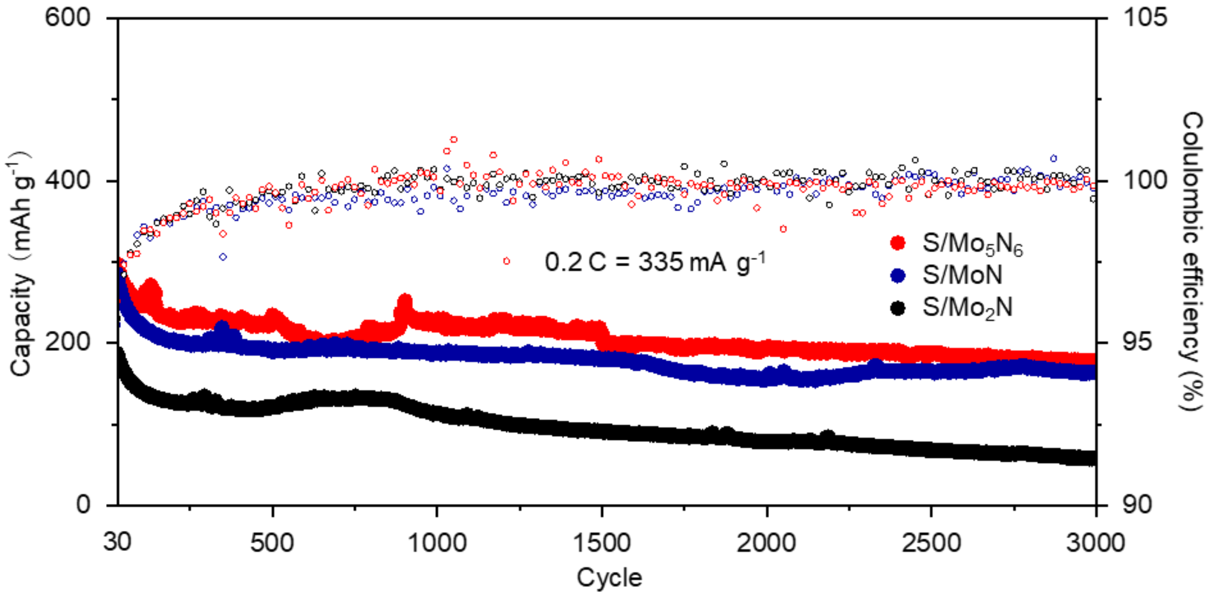

Supplementary Figure 9. Cycling performance and CE of the three sulfur electrodes at 0.2 C following 30 cycles of rating test under various rates.

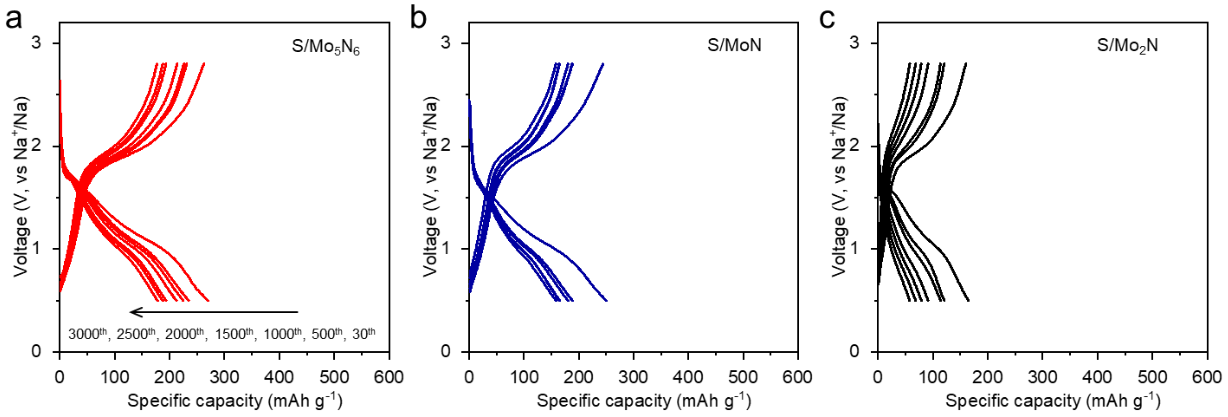

Supplementary Figure 10. a-c, Discharge/charge curves of the three sulfur electrodes at 0.2 C following the rating test.

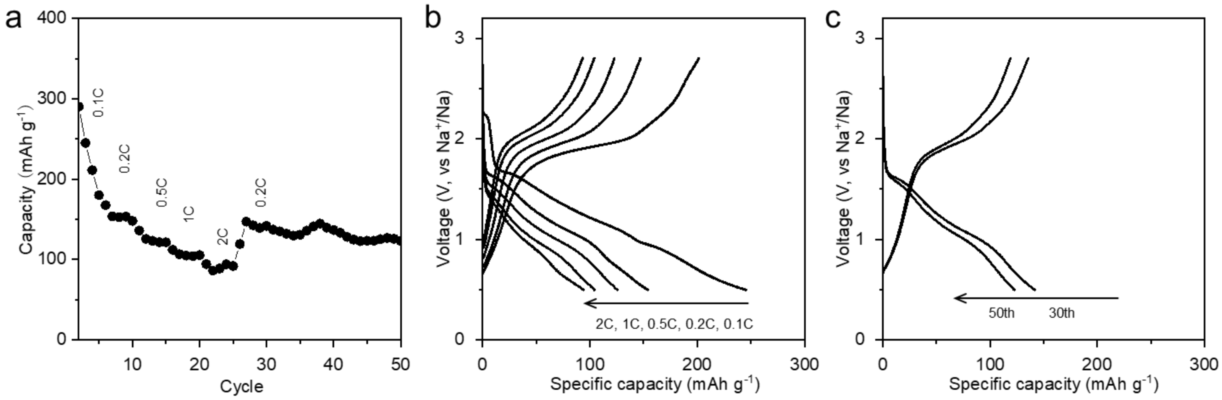

Supplementary Figure 11. a, Rating test for S/C electrode. Five cycles were recorded for each rate stepping from 0.1 to 2 C, and a following cycling test was conducted under 0.2 C for 20 cycles. The S/C electrode exhibited discharge capacities of 290, 150, 120, 105 and 88 mAh g^{-1} when cycled at, respectively, 0.1, 0.2, 0.5, 1 and 2 C (1 C = 1675 mA g^{-1}). When the specific current was switched back to 0.2 C, a low discharge capacity of 147 mAh g^{-1} was delivered. **b,** Discharge/charge curves for the S/C electrode under rates from 0.1 to 2 C. **c,** Discharge/charge curves for S/C electrode at 0.2 C following 30 cycles of rating test.

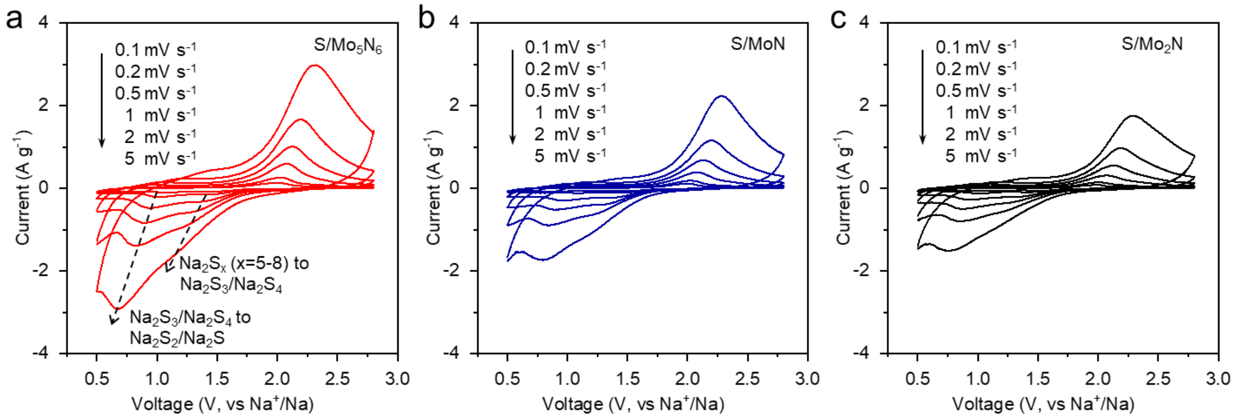

Supplementary Figure 12. a-c, CV curves of S/Mo_5N_6, S/MoN and S/Mo_2N with scan rates of 0.1 - 5 mV s^{-1}. The peak at ~1.5 to 1.2 V corresponds to the conversion of soluble Na_2S_x (x = 5-8) to soluble Na_2S_3/Na_2S_4. This result is consistent with reported the carbonate-based electrolyte system, whilst the tetra ethylene glycol dimethylether (TEGDME-based) electrolyte system generally exhibits a Na_2S_x (x = 5-8) reduction potential at ~ 2.0 V.[1] The peak at ~1.0 to 0.7 V corresponds to conversion of soluble Na_2S_3/Na_2S_4 to insoluble Na_2S_2/Na_2S.

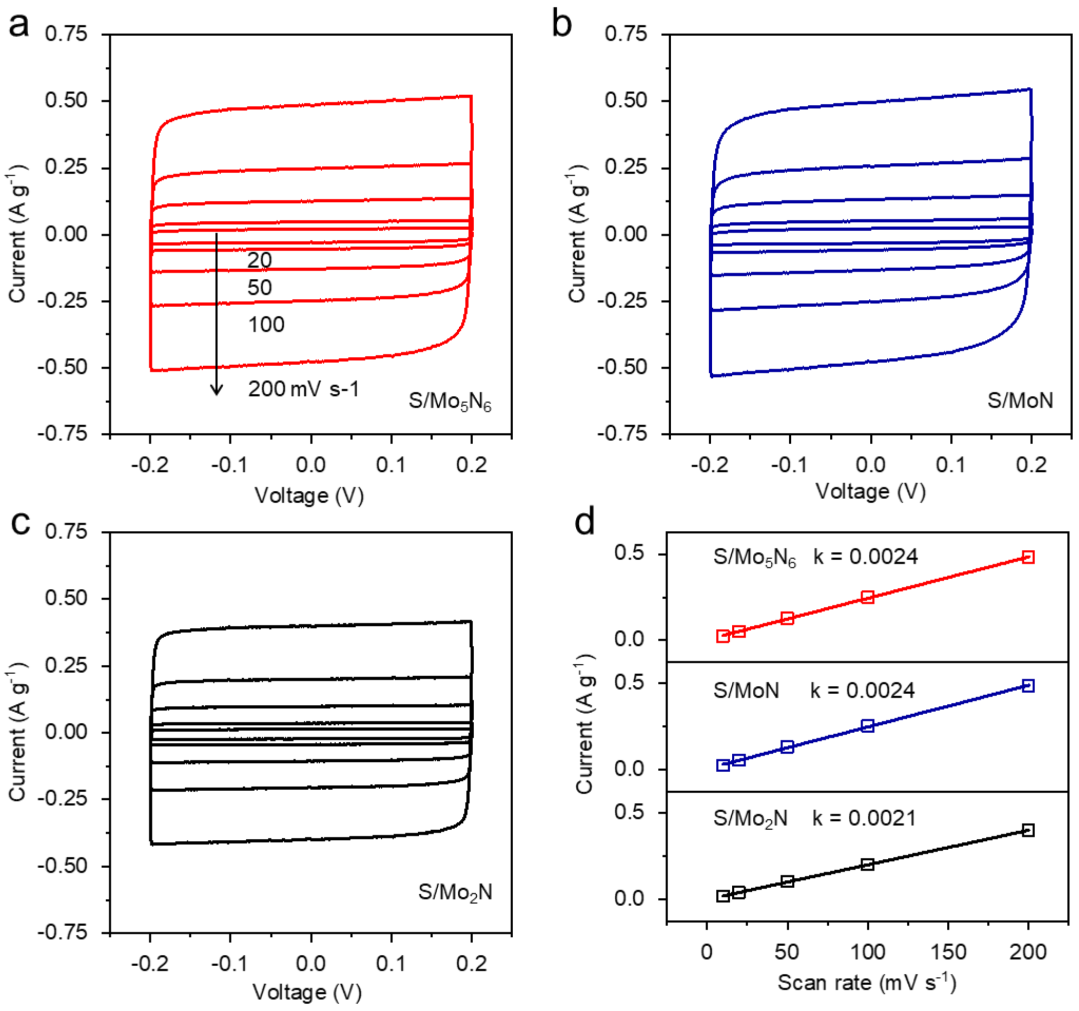

Supplementary Figure 13. CV curves of **a,** S/Mo_5N_6, **b,** S/MoN and **c,** S/Mo_2N symmetrical cells collected in the voltage range of -0.2 – 0.2 V with various scan rates. **d,** Scan rate dependence of the specific currents at 0.01 V. The surface area of the electrode (A) could be different due to the different morphologies of the Mo_5N_6, MoN and Mo_2N. To exclude the effect of electrode surface area on the sodium ion diffusion, we normalized the experimental i_p-$v^{1/2}$ slopes according to the electrochemically active surface areas (ECSAs) of S/Mo_5N_6, S/MoN and S/Mo_2N electrodes. Therefore, a series of CV tests under different scan rates from 10 to 200 mV s^{-1} were conducted to investigate the ECSAs of the three sulfur electrodes.

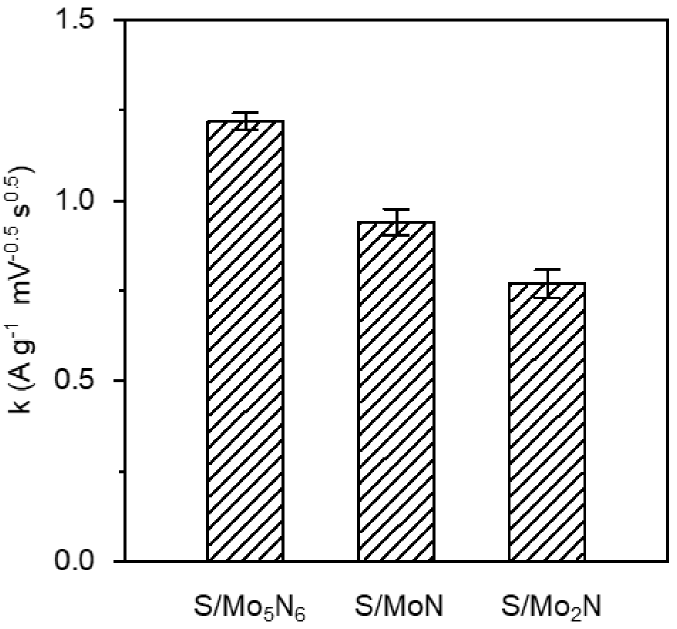

Supplementary Figure 14. Slopes of the peak current (i_p) and the square root of the scan rate ($\nu^{1/2}$) of the three sulfur electrodes, where A (electrode surface) is normalized based on ECSAs determined by CV tests. The error bars represent errors originating from the relative errors of the ECSAs.

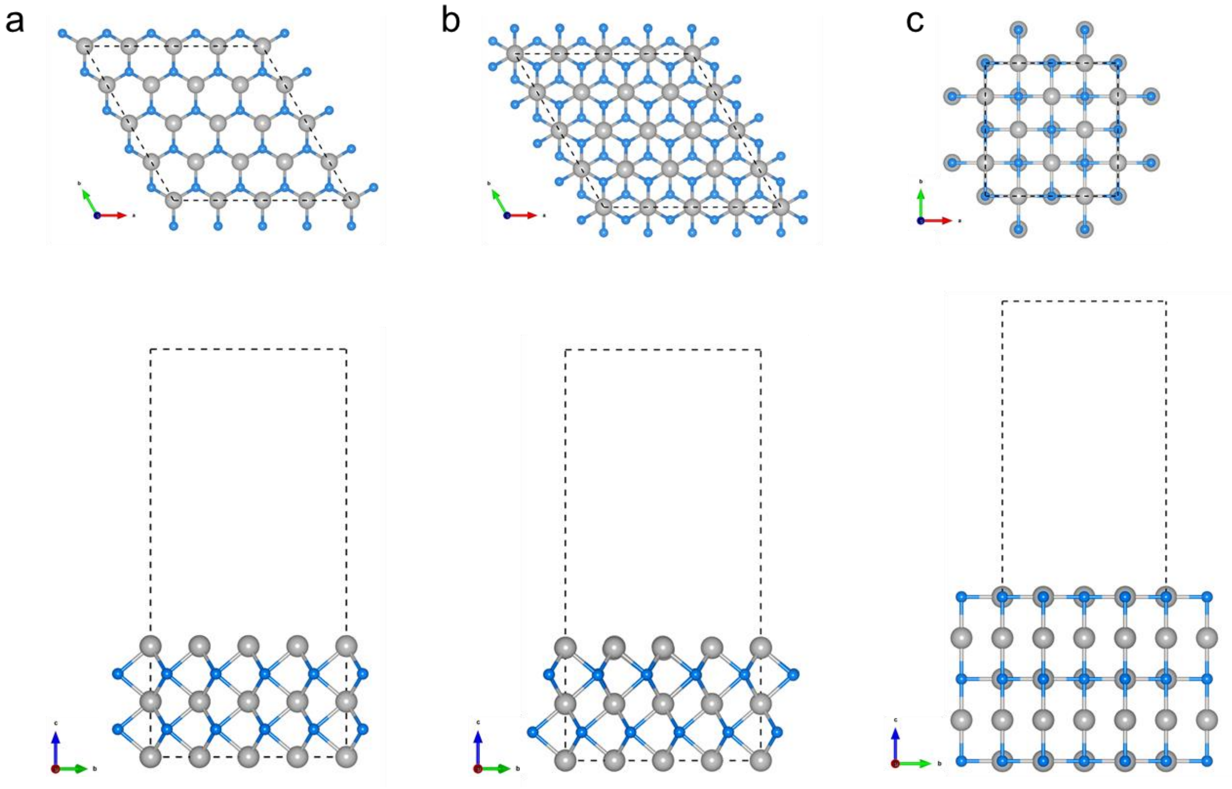

Supplementary Figure 15. a-c, Computational models for Mo_5N_6, MoN and Mo_2N, respectively. Color code is the same as for Figure. 1f.

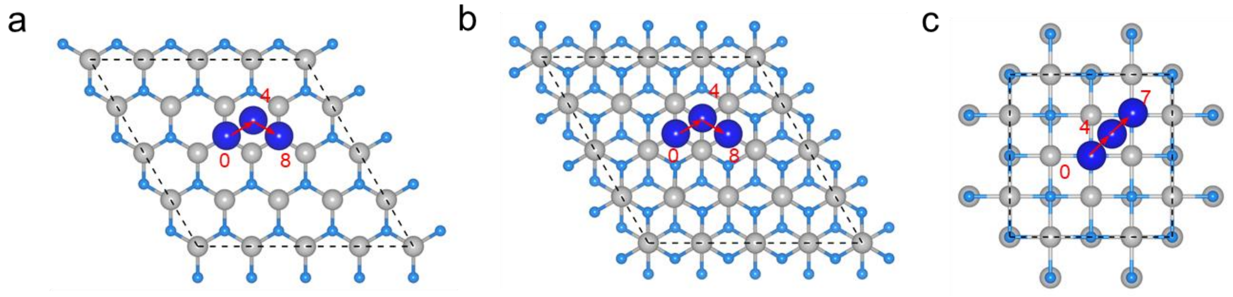

Supplementary Figure 16. a-c, Top view schematic of corresponding sodium ion diffusion pathways for Mo_5N_6, MoN and Mo_2N, respectively. Color code is the same as for the Figure. 5c.

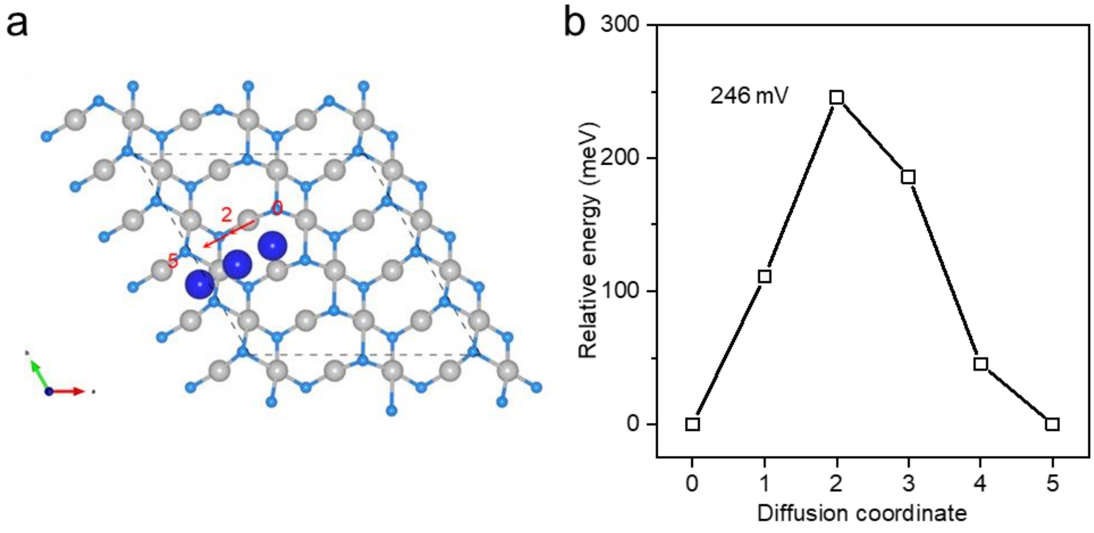

Supplementary Figure 17. a, Top view schematics of corresponding sodium ion diffusion pathway for the Mo_2N (1 1 1). Color code is the same as for the Figure. 5c. **b,** Energy profiles of the sodium ions diffusion on the Mo_2N (1 1 1).

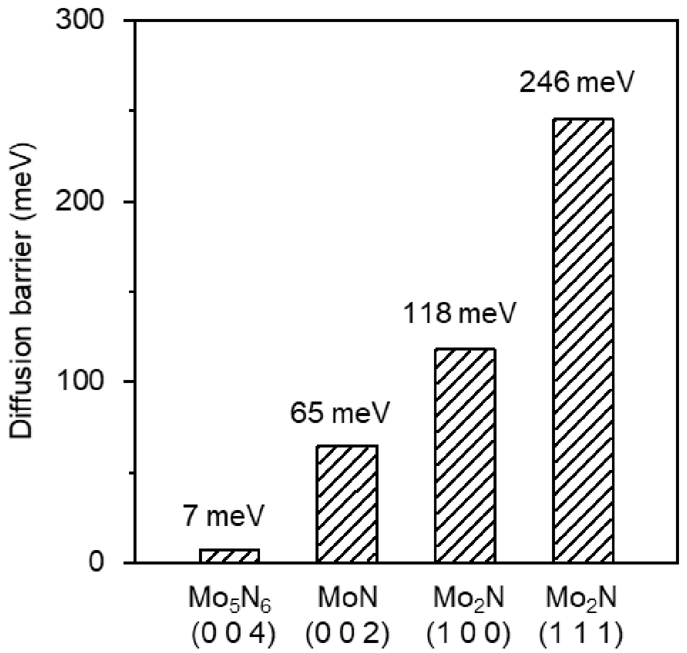

Supplementary Figure 18. Diffusion barriers of the sodium ions on the selected facets.

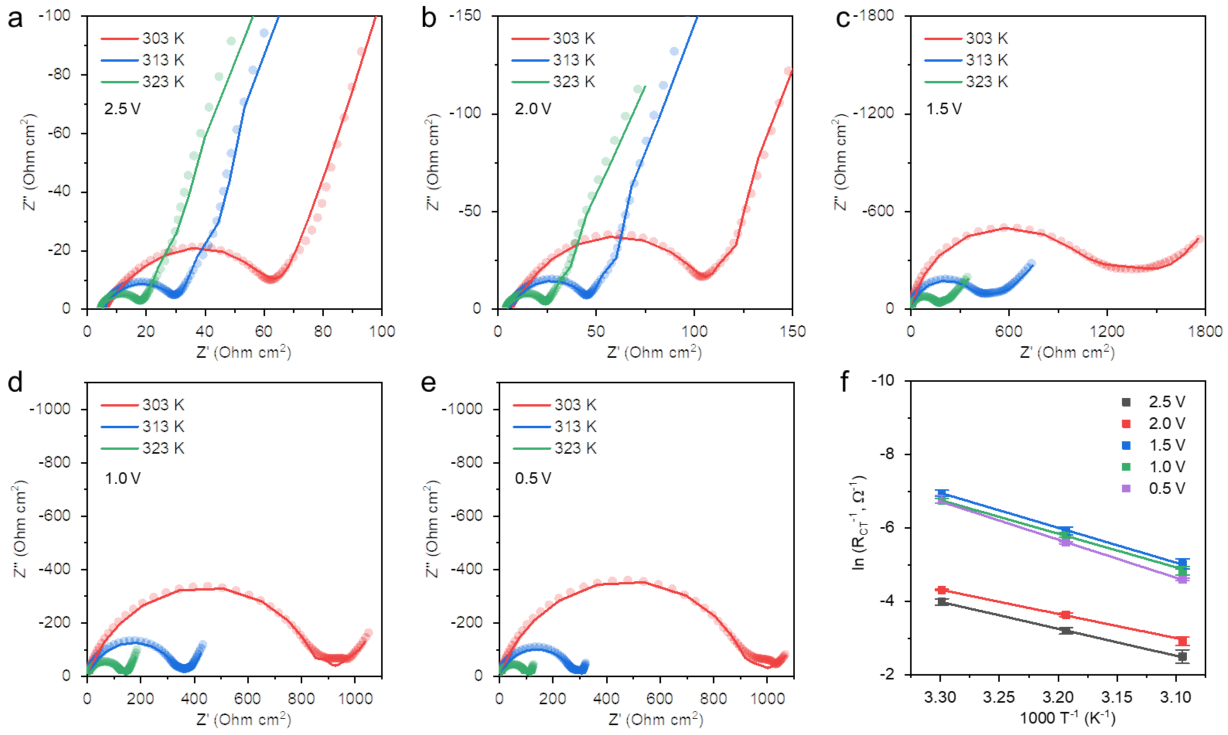

Supplementary Figure 19. a-e, EIS curves of the S/C electrodes at various voltages and temperatures. The raw impedance data and the fitted data is shown as symbols and lines. **f,** Arrhenius plots for charge transfer resistance (R_{ct}). The values of R_{ct} were obtained by fitting using equivalent circuit shown in the inset of Figure. 3a. The error bars in panel f represent relative errors of the fitted R_{ct} values.

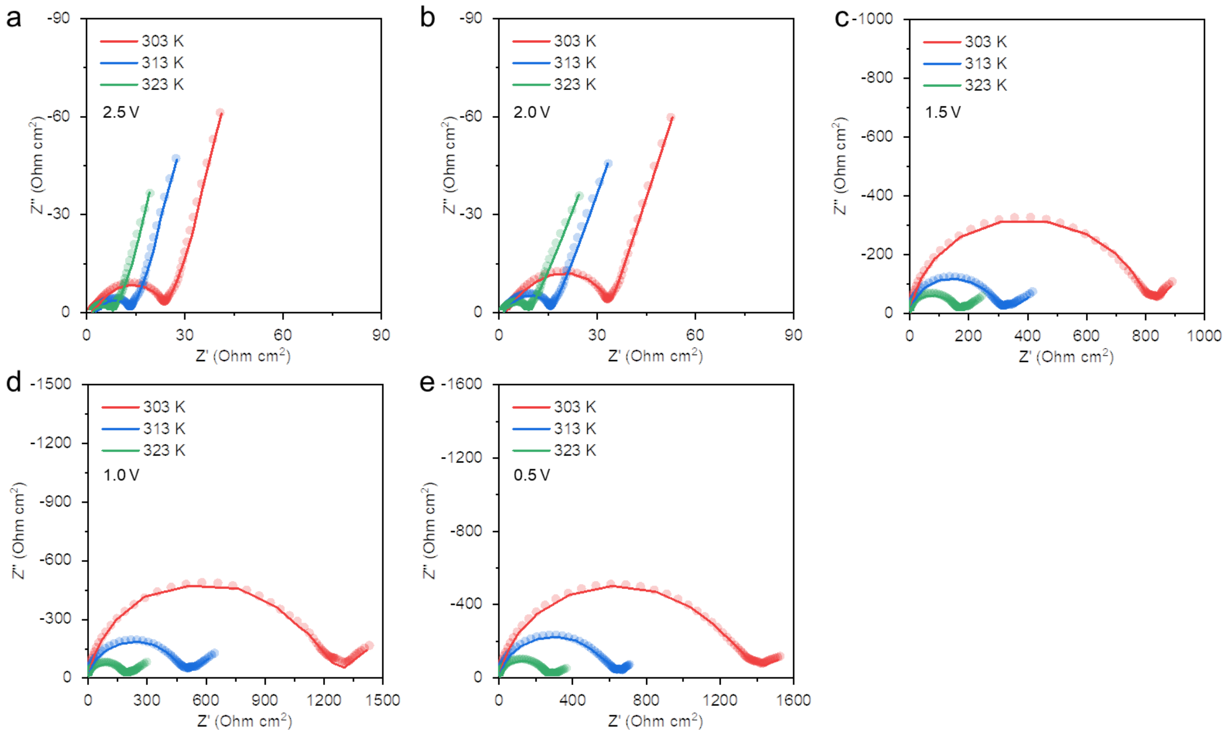

Supplementary Figure 20. a-e, EIS curves of the S/Mo_5N_6 electrodes at various voltages and temperatures. The raw impedance data and the fitted data is shown as symbols and lines.

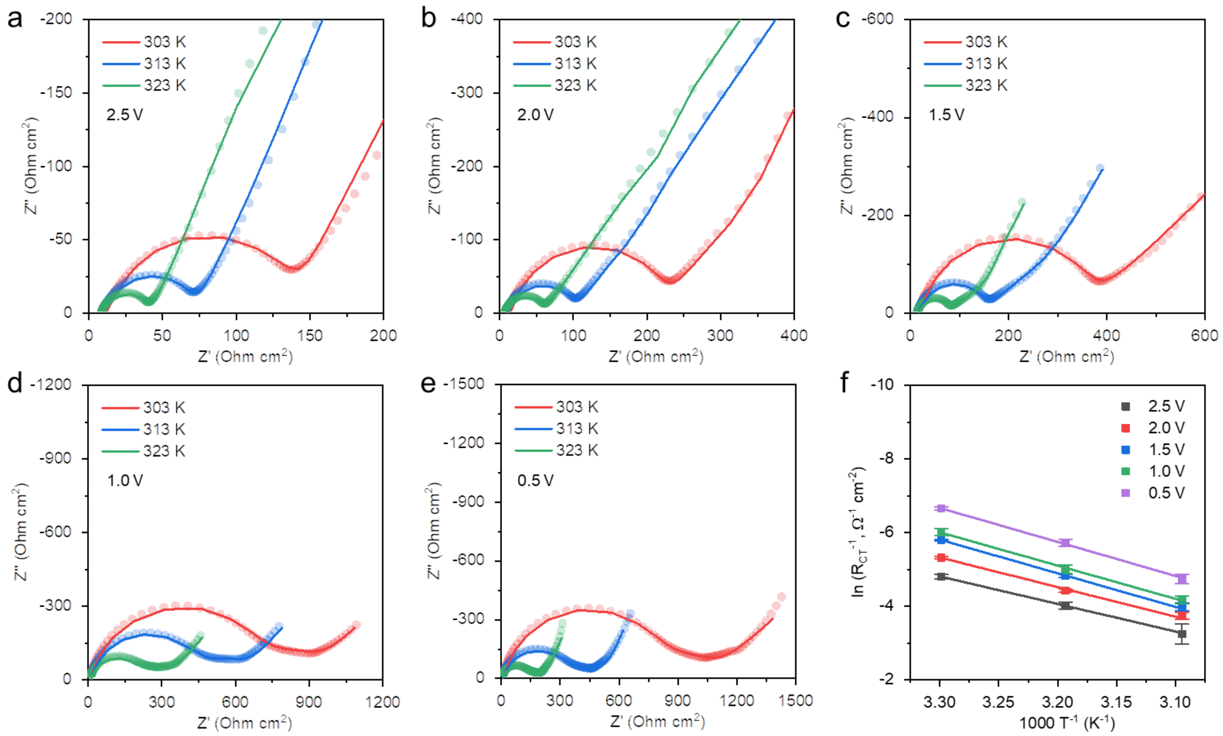

Supplementary Figure 21. a-e, EIS curves of the S/ MoN electrodes at various voltages and temperatures. The raw impedance data and the fitted data is shown as symbols and lines. **f,** Arrhenius plots for R_{ct}. The error bars in panel f represent relative errors of the fitted R_{ct} values. The values of R_{ct} were obtained by fitting using equivalent circuit shown in the inset of Figure. 3a. The error bars in panel f represent relative errors of the fitted R_{ct} values.

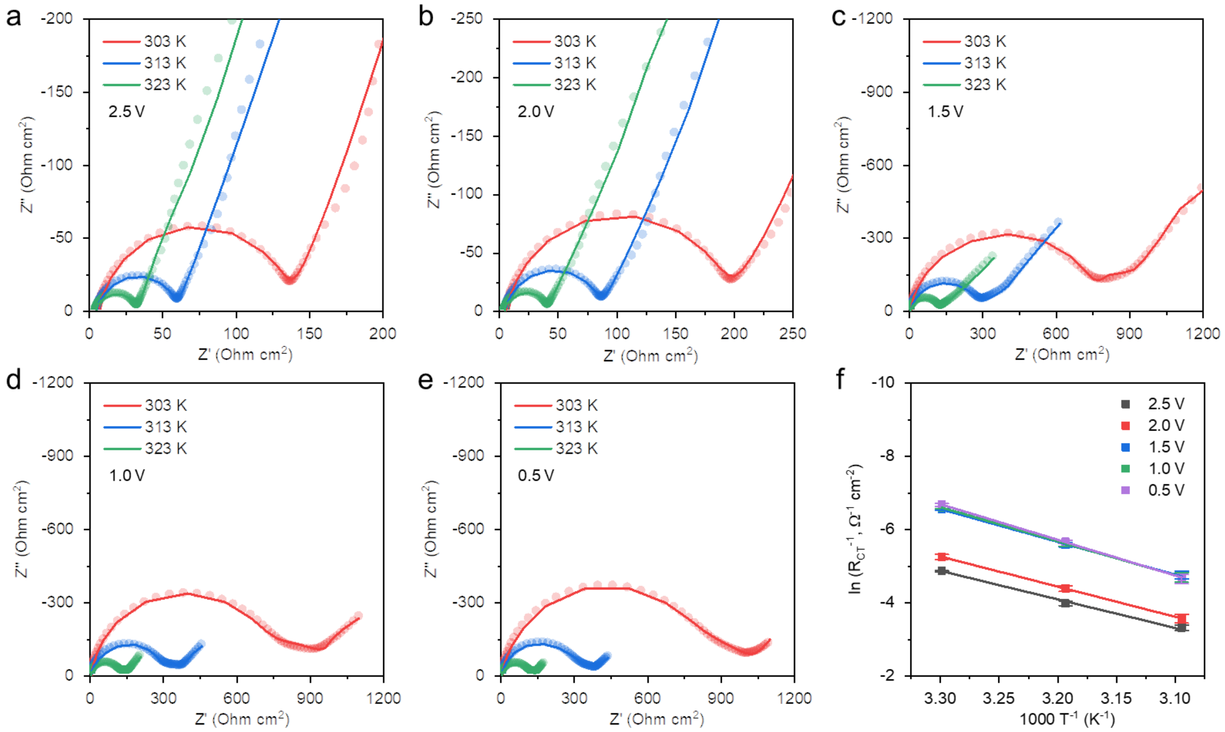

Supplementary Figure 22. a-e, EIS curves of the S/Mo_2N electrodes at various voltages and temperatures. The raw impedance data and the fitted data is shown as symbols and lines. **f,** Arrhenius plots for R_{ct}. The error bars in panel f represent relative errors of the fitted R_{ct} values. The values of R_{ct} were obtained by fitting using equivalent circuit shown in the inset of Figure. 3a. The error bars in panel f represent relative errors of the fitted R_{ct} values.

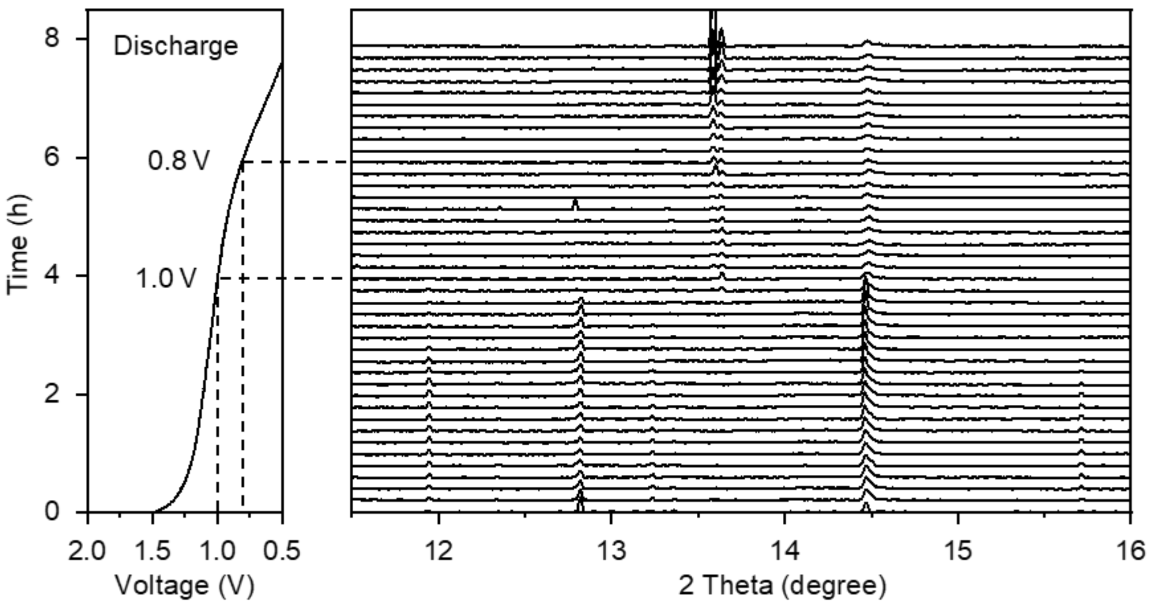

Supplementary Figure 23. In-situ synchrotron XRD patterns of Na_2S electrodeposition in S/Mo_5N_6.

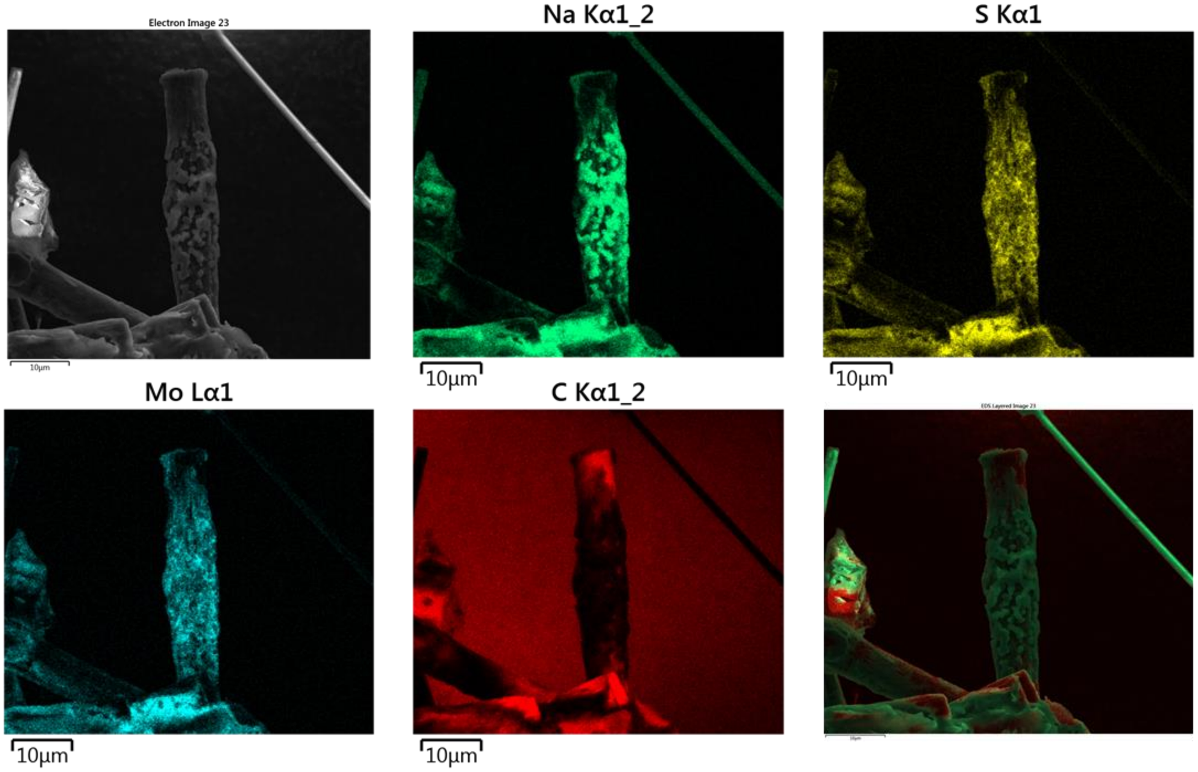

Supplementary Figure 24. SEM image and corresponding EDS elemental maps of electrodeposition test for the CP/Mo_5N_6 electrode.

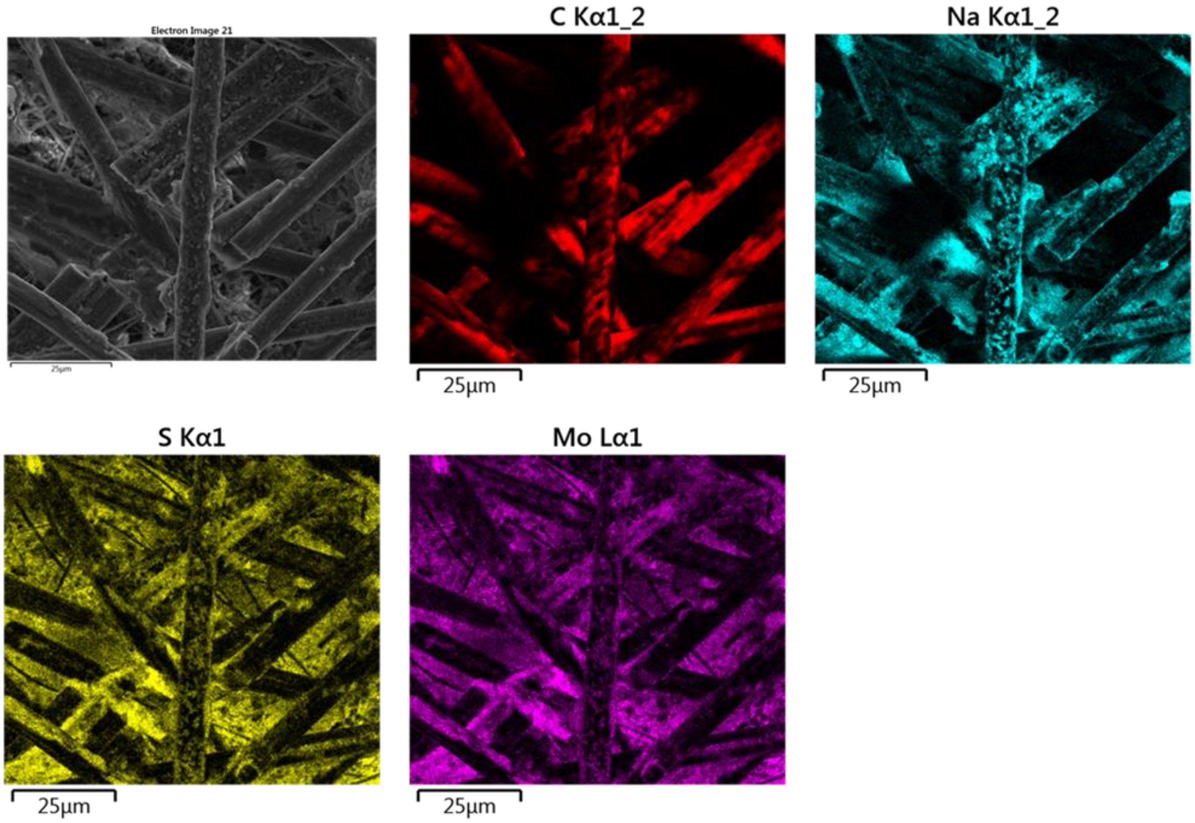

Supplementary Figure 25. SEM image and corresponding EDS elemental maps of electrodeposition test for the CP/MoN electrode.

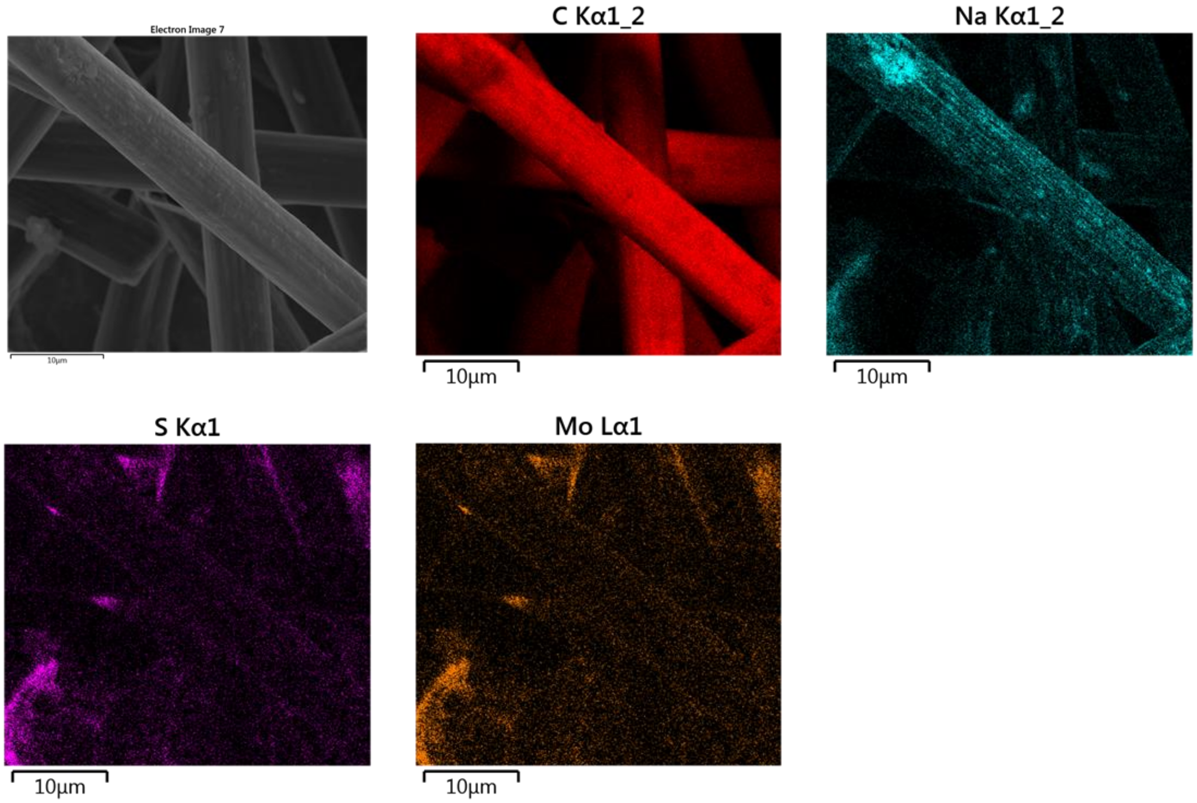

Supplementary Figure 26. SEM image and corresponding EDS elemental maps of electrodeposition test for the CP/Mo_2N electrode.

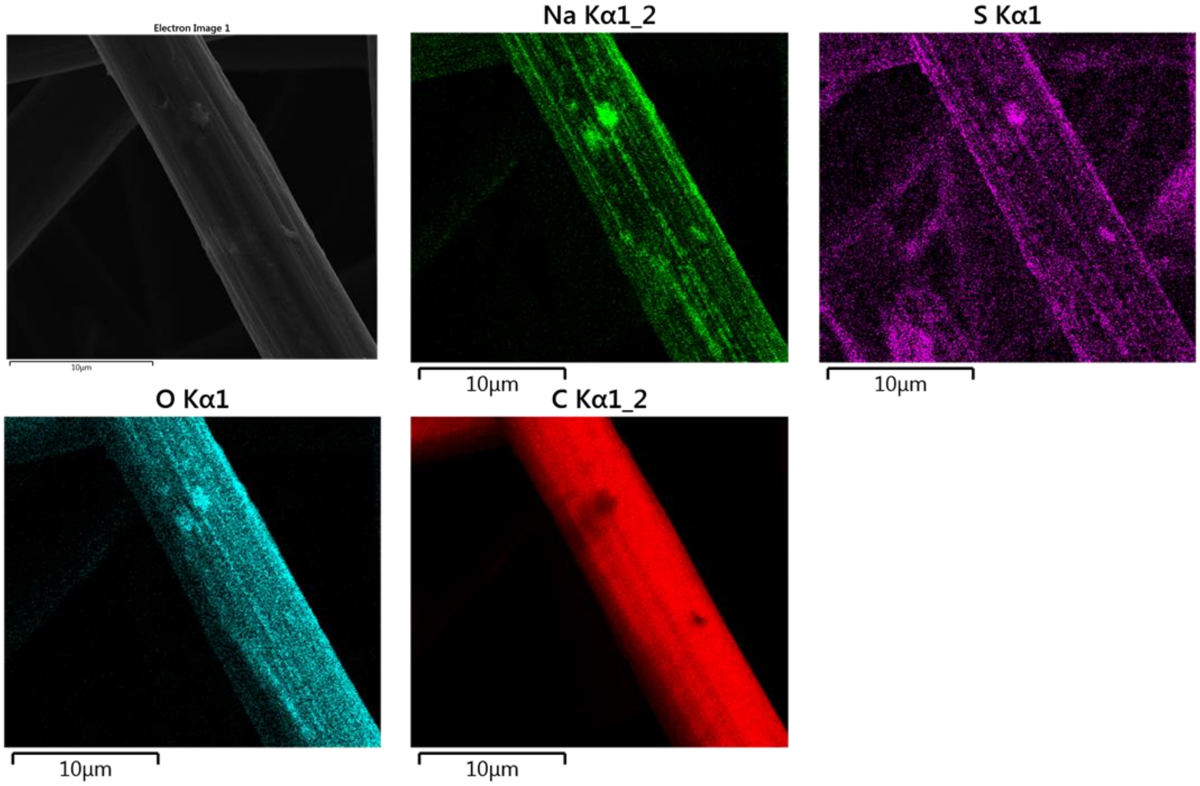

Supplementary Figure 27. SEM image and corresponding EDS elemental maps of electrodeposition test for the CP electrode.

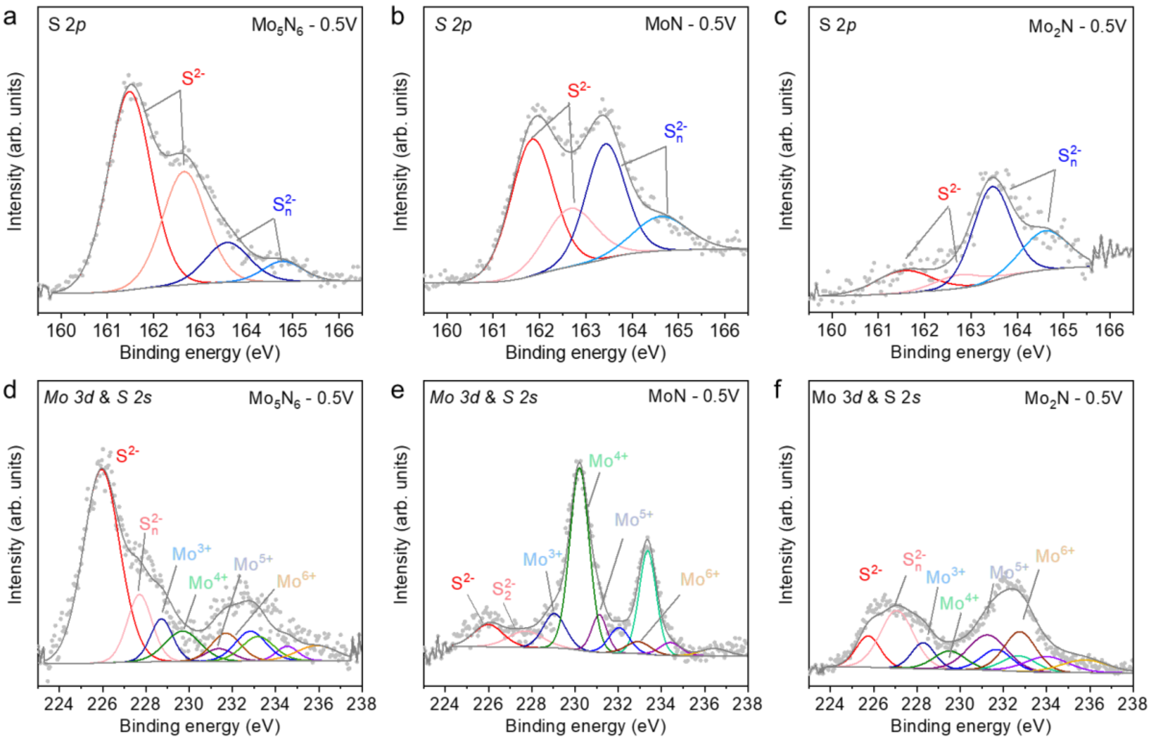

Supplementary Figure 28. S 2*p* XPS spectra of **a,** S/Mo_5N_6, **b,** S/MoN and **c,** S/Mo_2N sulfur cathodes at 0.5 V after the first discharge process under 0.1 C. Mo 3*d* and S 2*s* XPS spectra of **d,** S/Mo_5N_6, **e,** S/MoN and **f,** S/Mo_2N sulfur cathodes at 0.5 V after the first discharge process under 0.1 C. The S^{2-} and S_n^{2-} represent Na_2S and sodium polysulfides Na_2S_n (n=2-8) species, respectively.

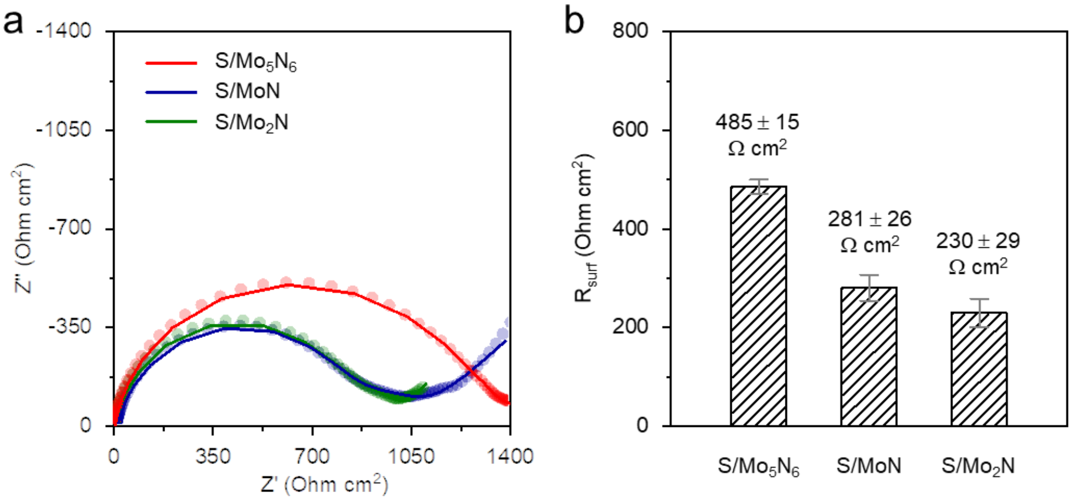

Supplementary Figure 29. a, EIS results of the three sulfur electrodes at the 0.5 V. The raw impedance data and the fitted data is shown as symbols and lines. **b,** The R_{surf} of the three sulfur electrodes at the 0.5 V. The error bars in panel b originate from relative errors of the fitted R_{surf} values.

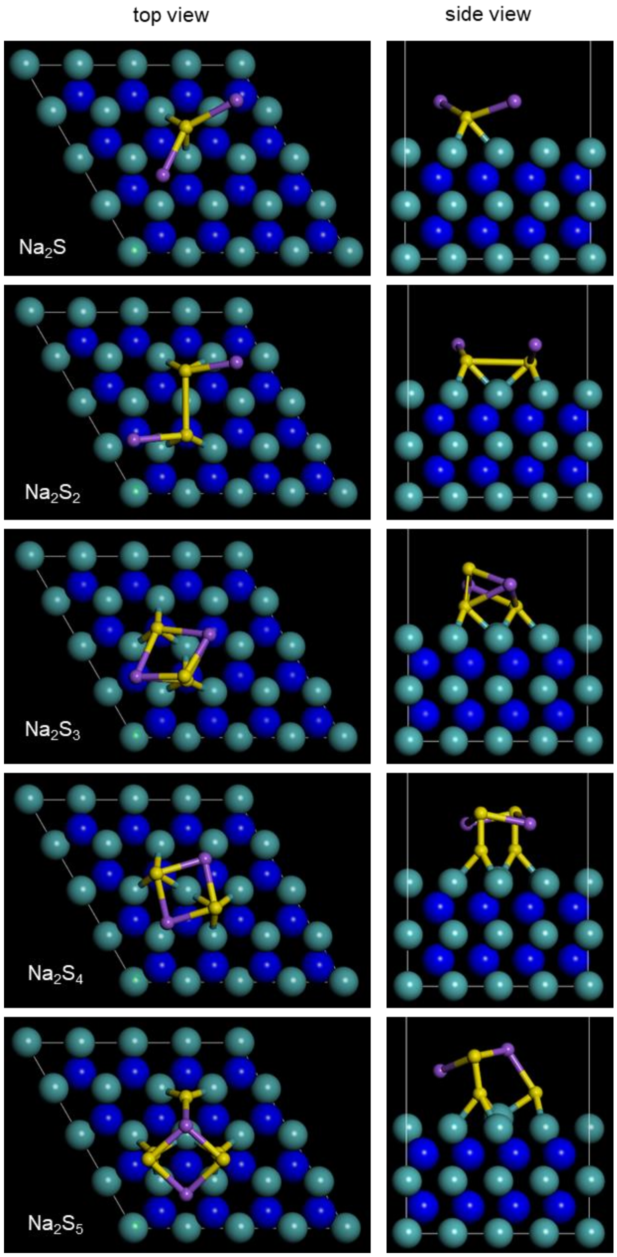

Supplementary Figure 30. Adsorption configurations for Na_2S_n (n = 1-5) on Mo_5N_6 (0 0 4) surface. The cyan-color, blue, yellow and purple spheres represent Mo, N, S and Na atoms, respectively.

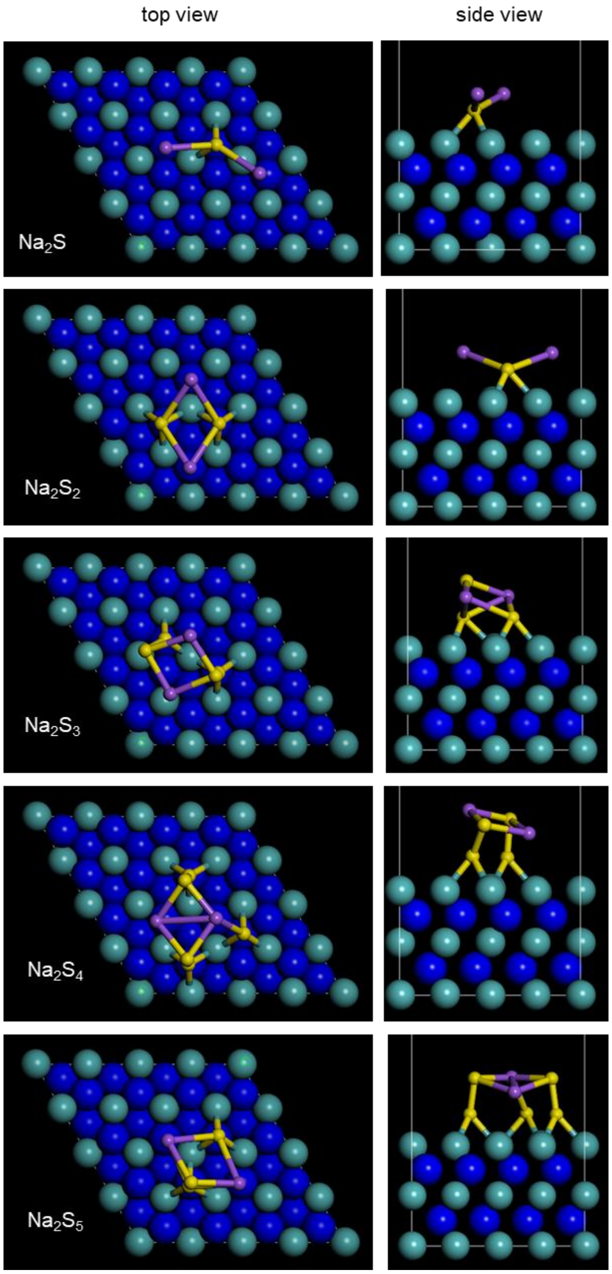

Supplementary Figure 31. Adsorption configurations for Na_2S_n (n = 1-5) on MoN (0 0 2) surface. Color code is the same as for Supplementary Figure. 32.

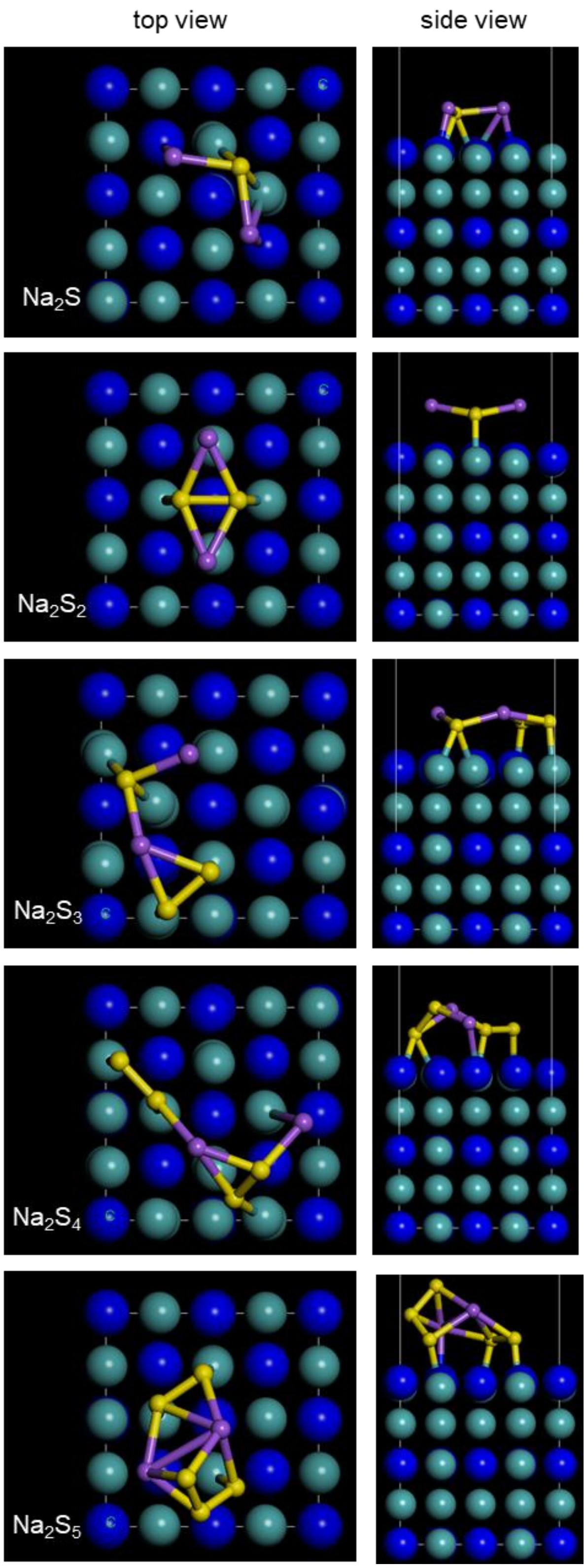

Supplementary Figure 32. Adsorption configurations for Na_2S_n (n = 1-5) on Mo_2N (1 0 0) surface. Color code is the same as for Supplementary Figure. 32.

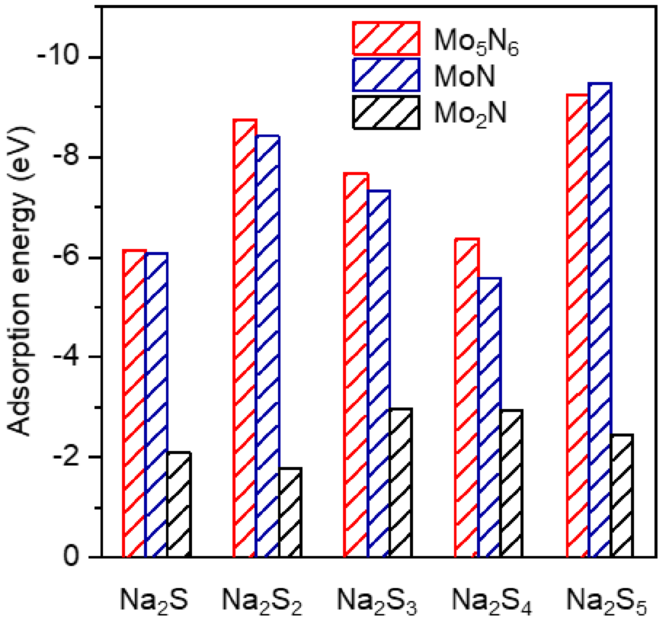

Supplementary Figure 33. Adsorption energies for Na_2S_n (n = 1-5) on the three molybdenum nitrides.

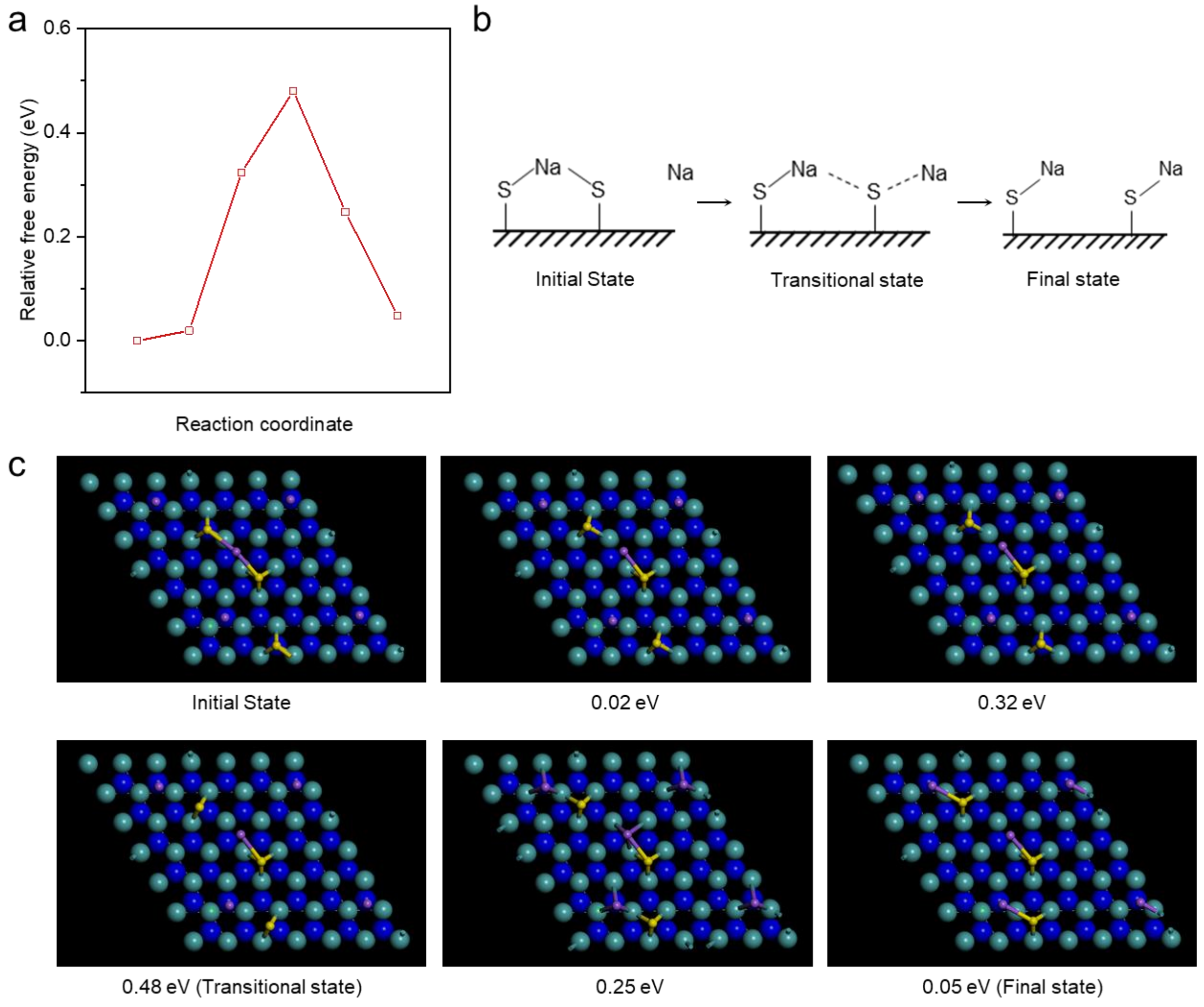

Supplementary Figure 34. a, Energy profile. **b,** Schematic of different states and **c,** Coordination obtained for each image on Mo_5N_6 (0 0 4) surface. Color code is the same as for Supplementary Figure. 32.

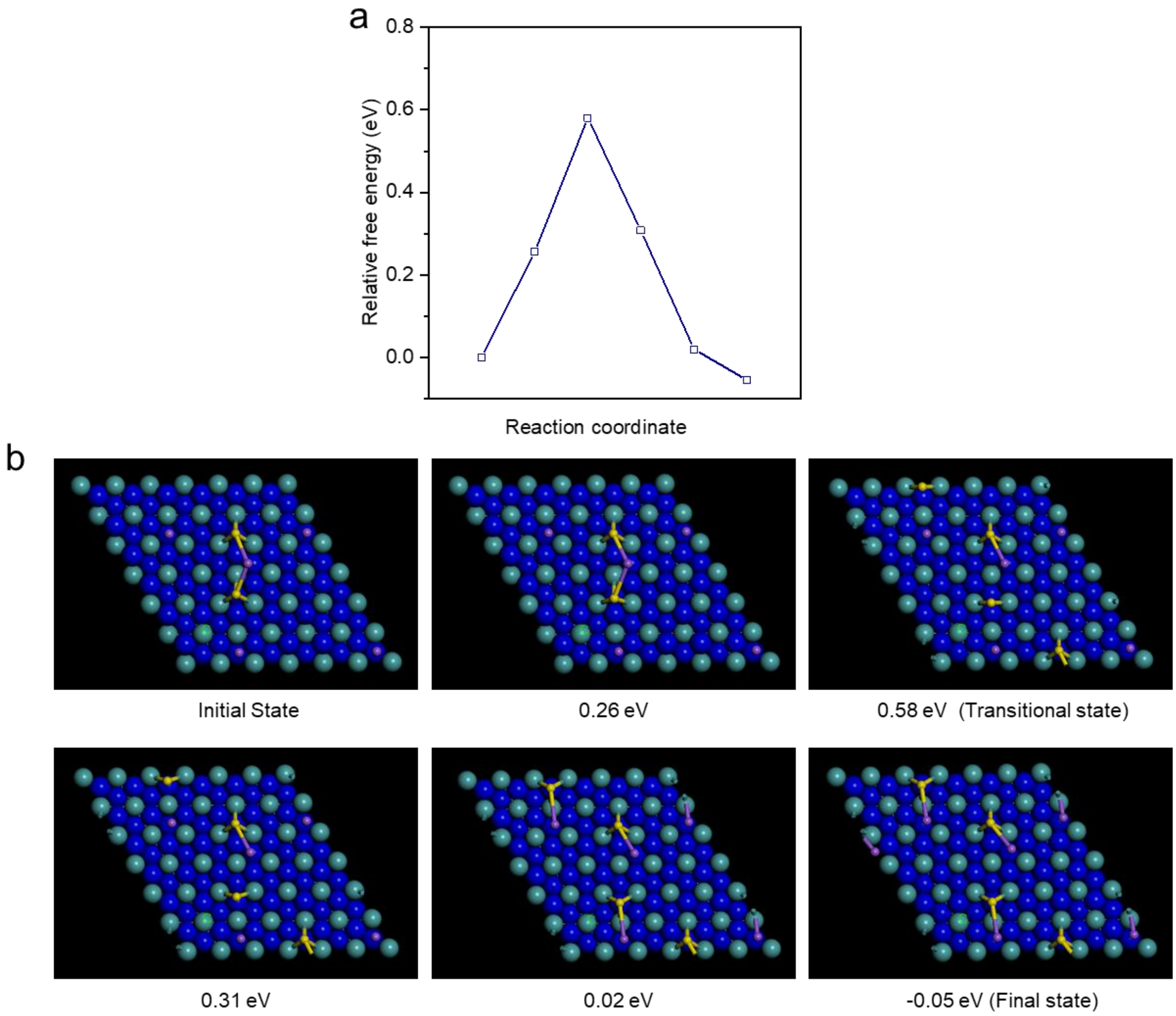

Supplementary Figure 35. a, Energy profile and **b,** Coordination obtained for each image on MoN (0 0 2) surface. Color code is the same as for Supplementary Figure. 32.

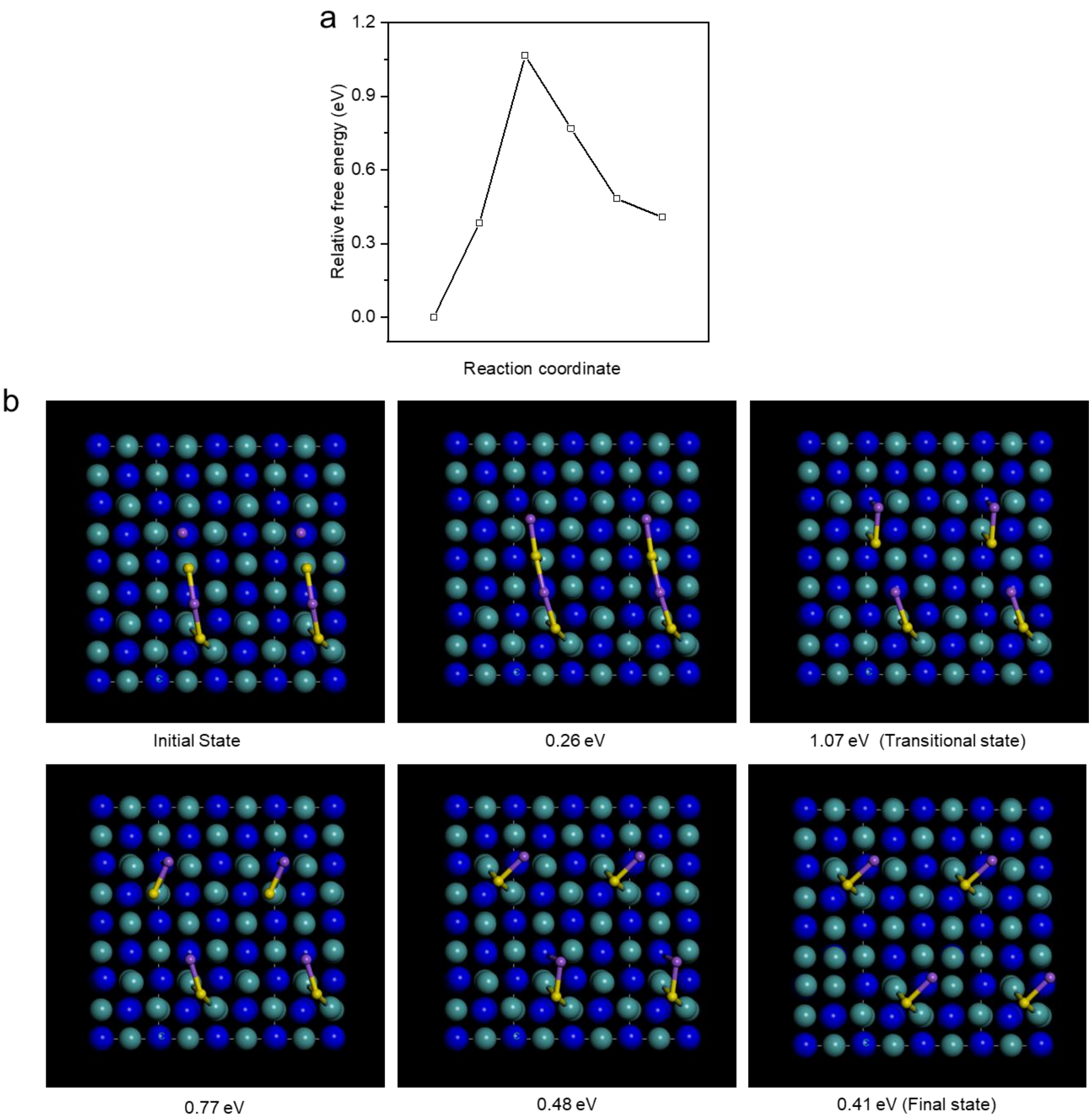

Supplementary Figure 36. a, Energy profile and **b,** Coordination obtained for each image on Mo_2N (1 0 0) surface. Color code is the same as for Supplementary Figure. 32.

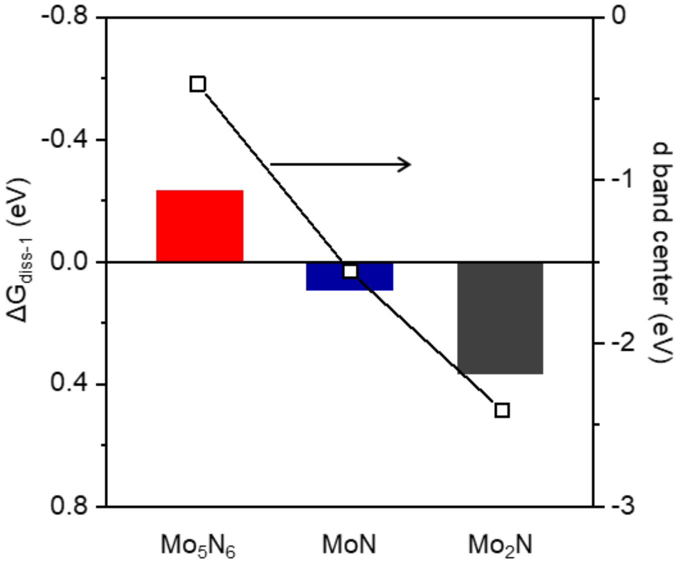

Supplementary Figure 37. Relationship between computed ΔG_{diss-1} value and the *d*-band center on the three molybdenum nitrides surfaces.

Supplementary Tables

Supplementary Table 1. Summary comparison of performance of S/Mo_5N_6 and recently reported room-temperature (RT) Na-S battery cathode materials.

**Sample**	**Testing temperature**	**Electrolyte type and amount (based on mass of the active material)**	**S content (%)**	**Rate (1C = 1675 mA g^{-1})**	**Cycles**	**Capacity decay (% per cycle)**	**Capacity (mA h g^{-1})**	**Ref.**
S/Mo_5N_6	RT, 303 K	1 M $NaClO_4$ in ethylene carbonate (EC)/propylene carbonate (PC) with 5 wt% fluoroethylene carbonate (FEC) ~11μL mg^{-1}	62.9	0.2<br>1	2970<br>10000	0.013<br>0.0064	298 (after rate tests)<br>180 (3000th cycle)<br>512<br>340 (100th cycle)<br>290 (1000th cycle)<br>186 (10000th cycle)	This work
S/Ni-MOF-2D	RT	1 M $NaClO_4$ in EC/PC with 5 wt% FEC 20μL mg^{-1}	48.6	1<br>2<br>0.2<br>2	1000<br>1000<br>970 (after rate)<br>rate	0.042<br>0.052<br>0.024	600 (1st cycle)<br>347 (1000th cycle)<br>500 (1st cycle)<br>241 (1000th cycle)<br>406<br>313<br>284	[2]
Na_2S_6/activated carbon cloth	RT	0.5 M $NaSO_3CF_3$ in TEGDME Not Available (NA)	NA	0.5<br>0.2<br>1<br>2	700<br>50<br>rate<br>rate	0.03<br>0.23	866 (1st cycle)<br>678 (700th cycle)<br>982 (1st cycle)<br>867 (50th cycle)<br>771<br>592	[3]
S/Fe clusters on hollow carbon spheres	RT	1 M $NaClO_4$ in EC/PC with 5 wt% FEC 10μL mg^{-1}	40	0.06<br>0.6<br>1.19<br>2.99	1000<br>rate<br>rate<br>rate	0.061	1023 (1st cycle)<br>394 (1000th cycle)<br>313<br>269<br>220	[4]

S/conductive carbon	RT	1 M $NaClO_4$ in EC/PC<br>NA	63	0.1<br>0.2<br>1	100<br>200<br>rate	0.071<br>0.17	700 (1st cycle)<br>650 (100th cycle)<br>525 (1st cycle)<br>350 (200th cycle)<br>200	5
S/Co cluster on carbon	RT	1 M $NaClO_4$ in EC/PC with 5 wt% FEC<br>NA	47	~ 0.06<br>0.6<br>1.19<br>2.99	600<br>rate<br>rate<br>rate	0.088	2075 (1st cycle)<br>1081 (2nd cycle)<br>508 (600th cycle)<br>313<br>269<br>220	6
Hollow Na_2S	RT	1 M $NaPF_6$ in diethylene glycol dimethyl ether (DIGLYME) and 1,3-dioxolane (DOL) with 0.08 M of Na_2S and P_2S_5 as additives.<br>~35μL mg^{-1}	NA	1.25<br>0.84	100<br>100	0.47<br>0.4	750 (1st cycle)<br>400 (100th cycle)<br>1000 (1st cycle)<br>600 (100th cycle)	7
S/hollow carbon	RT	1 M $NaClO_4$ in EC/PC with 5 wt% FEC<br>NA	71.2	2<br>0.1<br>1<br>2	400<br>50<br>rate<br>rate	0.044	315 (1st cycle)<br>260 (400th cycle)<br>1200 (1st cycle)<br>760 (50th cylce)<br>490<br>230	8
S/carbon microspheres	RT	1 M $NaClO_4$ in EC/PC with 5 wt% FEC<br>NA	34	0.06<br>1.19	350<br>rate	~ 0.21	1100 (1st cycle)<br>700 (2nd cycle)<br>300 (350th cycle)<br>60	9
S/carbon fiber cloth	RT	1.5 M $NaClO_4$ and 0.2 M $NaNO_3$ in tetra ethylene glycol dimethyl ether (TEGDME)<br>10μL mg^{-1}	24.4	0.1<br>1	300<br>rate	0.3	390 (1st cycle)<br>240 (2nd cycle)<br>120 (300th cycle)<br>50	10

S/micropo rous carbon	RT	1 M $NaPF_6$ and 0.25 M $NaNO_3$ in TEGDME<br>NA	35	1<br>0.1	1500<br>250	0.018<br>0.1	420 (1st cycle)<br>306 (1500th cycle)<br>930 (1st cycle)<br>420 (250th cycle)	11
S/hollow carbon	RT	1 M $NaClO_4$ in EC/PC with 5 wt% FEC<br>NA	46	0.06<br>0.6<br>1.19<br>2.99	200<br>rate<br>rate<br>rate	~ 0.38	1200 (1st cycle)<br>300 (200th cycle)<br>305<br>174<br>127	12
S/carboniz ed ZIF-8	RT	1 M $NaClO_4$ in TEGDME<br>NA	50	0.2<br>1<br>2	250<br>rate<br>rate	0.17	1400 (1st cycle)<br>873 (2nd cycle)<br>500 (250th cycle)<br>480<br>210	13
S/carbon	RT	1 M $NaClO_4$ in EC and dimethyl carbonate (DMC)<br>NA	18.13	~ 0.05<br>0.2	900<br>rate	0.054	1000 (1st cycle)<br>517 (900th cycle)<br>380	14
S/micropo rous carbon	RT	1 M $NaClO_4$ in EC and diethyl carbonate (DEC) or in TEGDME or in EC and PC<br>~80µL mg^{-1}	47	0.1<br>0.5	50<br>100	0.31<br>0.31	1614 (1st cycle)<br>800 (50th cycle)<br>1450 (1st cycle)<br>866 (2nd cycle)<br>600 (100th cycle)	15
S/Nafion @separato r	RT	1 M $NaClO_4$ in TEGDME<br>NA	42.5	0.1	20	0.63	400 (1st cycle)<br>350 (20th cycle)	16

Supplementary Table 2. Relative errors (%) of the fitted R_{ct} values for the four sulfur electrodes at varying voltages and temperatures.

		2.5V	2.0 V	1.5 V	1.0 V	0.5 V
S/C	303 K	8.02	2.13	7.97	6.13	9.43
S/C	313 K	8.33	5.10	9.98	6.45	3.07
S/C	323 K	17.09	10.53	13.04	10.81	3.73
S/Mo_5N_6	303 K	6.75	5.08	4.08	1.82	4.34
S/Mo_5N_6	313 K	6.80	6.67	4.50	1.29	5.36
S/Mo_5N_6	323 K	17.14	6.49	2.16	3.66	16.46
S/MoN	303 K	6.72	2.17	2.21	8.33	4.60
S/MoN	313 K	9.15	4.88	3.98	10.23	8.22
S/MoN	323 K	24.08	9.21	10.41	10.53	11.94
S/Mo_2N	303 K	1.98	6.79	2.58	2.76	3.66
S/Mo_2N	313 K	5.86	7.59	5.78	7.19	4.26
S/Mo_2N	323 K	7.41	11.33	10.10	12.04	11.72

Supplementary Table 3. Activation energies (eV) with errors for the four sulfur electrode at varying voltage.

Voltage	S/C	S/Mo_5N_6	S/MoN	S/Mo_2N
2.5 V	0.63 ± 0.01	0.53 ± 0.01	0.64 ± 0.01	0.67 ± 0.03
2.0 V	0.57 ± 0.02	0.57 ± 0.01	0.69 ± 0.04	0.71 ± 0.01
1.5 V	0.82 ± 0.01	0.60 ± 0.02	0.79 ± 0.02	0.77 ± 0.02
1.0 V	0.79 ± 0.03	0.73 ± 0.02	0.78 ± 0.02	0.80 ± 0.01
0.5 V	0.90 ± 0.03	0.74 ± 0.01	0.80 ± 0.02	0.84 ± 0.02

Supplementary Table 4. Adsorption energies (eV) for Na_2S_5, Na_2S_4, Na_2S_3, Na_2S_2 and Na_2S on three molybdenum nitrides surfaces.

	Mo_5N_6 (0 0 4)	MoN (0 0 2)	Mo_2N (1 0 0)
Na_2S	-6.14	-6.06	-2.10
Na_2S_2	-8.75	-8.41	-1.78
Na_2S_3	-7.67	-7.33	-2.96
Na_2S_4	-6.36	-5.58	-2.93
Na_2S_5	-9.23	-9.45	-2.44

Supplementary Table 5. Frequencies (meV) of the initial, transition, and final states for Na_2S_2 dissociation on three molybdenum nitrides surfaces. i refers to an imaginary frequency. TS refers to transitional state.

Mo_5N_6 (0 0 4)			MoN (0 0 2)			Mo_2N (1 0 0)		
$*Na_2S_2$	TS*	$*NaS_2$ +*Na	$*Na_2S_2$	TS*	$*NaS_2$ +*Na	$*Na_2S_2$	TS*	$*NaS_2$ +*Na
41.34	42.14	44.15	41.11	42.07	43.73	44.09	38.14	41.53
40.12	41.10	41.26	39.93	39.94	40.40	32.32	30.07	29.42
28.78	29.11	27.91	28.12	28.93	27.96	29.11	27.48	28.51
27.50	27.46	27.74	27.54	28.04	27.44	28.19	21.37	26.88
27.26	27.32	27.48	27.38	27.21	27.09	21.62	19.04	18.70
26.03	22.22	27.46	26.66	21.92	26.77	18.85	18.14	18.47
22.10	21.82	22.02	22.23	21.50	21.98	15.59	16.11	18.21
21.61	10.04	21.64	21.84	11.36	21.52	15.24	12.68	17.36
9.91	9.19	10.60	11.56	10.08	11.09	13.21	9.98	13.41
9.54	5.67	9.38	10.67	5.55	9.46	10.30	8.48	9.81
8.36	4.59	5.31	5.81	4.15	5.57	7.11	6.01	7.69
5.34	24.61i	4.53	3.75	22.20i	3.82	5.29	2.30i	5.53

Supplementary References

1. Wang Y*, et al.* Revitalising sodium–sulfur batteries for non-high-temperature operation: a crucial review. *Energy Environ Sci* **13**, 3848-3879 (2020).

2. Ye C*, et al.* Electron-State Confinement of Polysulfides for Highly Stable Sodium–Sulfur Batteries. *Adv Mater* **32**, 1907557 (2020).

3. Zhang S, Pollard TP, Feng X, Borodin O, Xu K, Li Z. Altering the Electrochemical Pathway of Sulfur Chemistry with Oxygen for High Energy Density and Low Shuttling in a Na/S Battery. *ACS Energy Lett* **5**, 1070-1076 (2020).

4. Zhang B-W*, et al.* Long-Life Room-Temperature Sodium–Sulfur Batteries by Virtue of Transition-Metal-Nanocluster–Sulfur Interactions. *Angew Chem In Ed* **58**, 1484-1488 (2019).

5. Vijaya Kumar Saroja AP, Muthusamy K, Sundara R. Strong Surface Bonding of Polysulfides by Teflonized Carbon Matrix for Enhanced Performance in Room Temperature Sodium-Sulfur Battery. *Adv Mater Interfaces* **6**, 1801873 (2019).

6. Zhang B-W*, et al.* Atomic cobalt as an efficient electrocatalyst in sulfur cathodes for superior room-temperature sodium-sulfur batteries. *Nat Commun* **9**, 4082 (2018).

7. Wang C*, et al.* Frogspawn‐Coral‐Like Hollow Sodium Sulfide Nanostructured Cathode for High‐Rate Performance Sodium–Sulfur Batteries. *Adv Energy Mater* **9**, 1803251 (2018).

8. Xia G*, et al.* Carbon Hollow Nanobubbles on Porous Carbon Nanofibers: An Ideal Host for High-Performance Sodium-Sulfur Batteries and Hydrogen Storage. *Energy Stor Mater* **14**, 314-323 (2018).

9. Zhang L*, et al.* Self-Assembling Hollow Carbon Nanobeads into Double-Shell Microspheres as a Hierarchical Sulfur Host for Sustainable Room-Temperature Sodium–Sulfur Batteries. *ACS Appl Mater Interfaces* **10**, 20422-20428 (2018).

10. Lu Q*, et al.* Freestanding carbon fiber cloth/sulfur composites for flexible room-temperature sodium-sulfur batteries. *Energy Stor Mater* **8**, 77-84 (2017).

11. Carter R, Oakes L, Douglas A, Muralidharan N, Cohn AP, Pint CL. A Sugar-Derived Room-Temperature Sodium Sulfur Battery with Long Term Cycling Stability. *Nano Lett* **17**, 1863-1869 (2017).

12. Wang Y-X*, et al.* Achieving high-performance room-temperature sodium-sulfur batteries with S@interconnected mesoporous carbon hollow nanospheres. *J Am Chem Soc* **138**, 16576-16579 (2016).

13. Chen Y-M*, et al.* A nitrogen doped carbonized metal–organic framework for high stability room temperature sodium–sulfur batteries. *J Mater Chem A* **4**, 12471-12478 (2016).

14. Fan L, Ma R, Yang Y, Chen S, Lu B. Covalent sulfur for advanced room temperature sodium-sulfur batteries. *Nano energy* **28**, 304-310 (2016).

15. Wei S*, et al.* A stable room-temperature sodium–sulfur battery. *Nat Commun* **7**, 11722 (2016).

16. Bauer I, Kohl M, Althues H, Kaskel S. Shuttle suppression in room temperature sodium–sulfur batteries using ion selective polymer membranes. *Chem Commun* **50**, 3208-3210 (2014).
